# How much flower‐rich habitat is enough for wild pollinators? Answering a key policy question with incomplete knowledge

**DOI:** 10.1111/een.12226

**Published:** 2015-07-02

**Authors:** LYNN V. DICKS, MATHILDE BAUDE, STUART P. M. ROBERTS, JAMES PHILLIPS, MIKE GREEN, CLAIRE CARVELL

**Affiliations:** ^1^ Department of ZoologyUniversity of CambridgeCambridgeU.K.; ^2^ Collegium Sciences et Techniques, LBLGC EA 1207, Université d'OrléansOrléansFrance; ^3^ School of Biological Sciences, University of BristolBristolU.K.; ^4^ Centre for Agri‐Environmental Research, School of Agriculture, Policy & Development, University of ReadingReadingU.K.; ^5^ Natural EnglandSomersetU.K.; ^6^ Natural EnglandWorcesterU.K.; ^7^ NERC Centre for Ecology and HydrologyOxfordshireU.K.

**Keywords:** Agri‐environment scheme, Apoidea, bee, farm, floral resources, landscape, policy window, pollen, pollination, pollinator

## Abstract

In 2013, an opportunity arose in England to develop an agri‐environment package for wild pollinators, as part of the new Countryside Stewardship scheme launched in 2015. It can be understood as a ‘policy window’, a rare and time‐limited opportunity to change policy, supported by a narrative about pollinator decline and widely supported mitigating actions. An agri‐environment package is a bundle of management options that together supply sufficient resources to support a target group of species. This paper documents information that was available at the time to develop such a package for wild pollinators. Four questions needed answering: (1) Which pollinator species should be targeted? (2) Which resources limit these species in farmland? (3) Which management options provide these resources? (4) What area of each option is needed to support populations of the target species? Focussing on wild bees, we provide tentative answers that were used to inform development of the package. There is strong evidence that floral resources can limit wild bee populations, and several sources of evidence identify a set of agri‐environment options that provide flowers and other resources for pollinators. The final question could only be answered for floral resources, with a wide range of uncertainty. We show that the areas of some floral resource options in the basic Wild Pollinator and Farmland Wildlife Package (2% flower‐rich habitat and 1 km flowering hedgerow), are sufficient to supply a set of six common pollinator species with enough pollen to feed their larvae at lowest estimates, using minimum values for estimated parameters where a range was available. We identify key sources of uncertainty, and stress the importance of keeping the Package flexible, so it can be revised as new evidence emerges about how to achieve the policy aim of supporting pollinators on farmland.

## Introduction: the policy opportunity

There has been substantial global concern among scientists, governments, businesses, and the public about observed declines in wild and managed pollinators (Potts *et al*., 2010; Dicks *et al*., 2012; Carvalheiro *et al*., 2013; Dicks, 2013). There is evidence of declines in abundance, or species richness, for some pollinator groups, of contracting distributions in many wild pollinator species, and of heightened colony losses in managed honey bees (Potts *et al*., 2010). These declines are usually attributed to multiple interacting causes, rather than one single cause (Vanbergen *et al*. 2013), although pesticide use has recently been prominent in the public and political discourse.

In response to this, several countries, including England, Wales, France, Brazil, and the U.S.A., have initiated strategic, national level policy initiatives on pollinators. The National Pollinator Strategy for England (Defra, 2014a) was developed between June 2013 when the intention was first announced, and November 2014, when the strategy was published. This period of policy formulation coincided with the development of England's policy responses to the reform of the European Union's Common Agricultural Policy (Pe'er *et al*., 2014), which has included the development of a new voluntary agri‐environment scheme for England, Countryside Stewardship (Defra, 2014b).

The coincidence of pollinator and agricultural policy formulation in England, combined with widespread public concern about pollinators, created what has been called a ‘policy window’ (Kingdon, 2003; Dudley, 2013). Policy windows are opportunities to develop specific ideas within policy, opened by the appearance of compelling problems or by ‘happenings in the political stream’ (Kingdon, 2003). They are short‐lived and infrequent, and when they occur, they represent a limited and valuable opportunity for a body of scientific knowledge to influence policy. In this case, the UK Government's Department for Environment, Food and Rural Affairs (Defra) and its statutory nature conservation agency for England (Natural England) were particularly keen to develop agri‐environment scheme elements with a strong focus on supporting wild pollinators, in order to make a significant contribution towards the National Pollinator Strategy. The 10‐year Strategy aims to deliver across five key areas, including ‘Supporting pollinators on farmland through the CAP’, with a key outcome to see ‘More, bigger, better, joined‐up, diverse and high‐quality flower‐rich habitats (including nesting places and shelter) supporting our pollinators across the country’ (Defra, 2014a).

One approach to targeting a taxon or component of farmland biodiversity using agri‐environment schemes that is considered to have been successful among policymakers is the ‘Farmland Bird Package’ of agri‐environment options for English arable and mixed farms (Winspear *et al*., 2010). This was developed by a partnership of Government and Non‐Governmental Organisations, based on identifying and enhancing the resources that are limiting declining farmland bird species in farmed landscapes (notably the availability of nesting habitats and year‐round food resources). The narrative that accompanies the Farmland Bird Package is that it worked because it was (i) simple and straightforward to implement, and (ii) built on an assessment of available evidence of what declining farmland birds need. It could, therefore, be expected to deliver, by providing sufficient appropriate resources to reverse the declines in focal species of farmland bird. Data to show that the Farmland Bird Package has actually increased populations of declining farmland bird species or increased the uptake of its specific agri‐environment options in the target areas, are not currently in the public domain. Analyses conducted by Natural England demonstrate that the package has had some success on both counts, and six target bird species are known to have responded positively to Higher Level Stewardship agreements that pre‐date full development of the Farmland Bird Package, but contained many of the same options (Bright *et al*., 2015). The policy opportunity in 2013 and 2014 was to take this apparently successful approach and apply it to wild pollinators, by developing a ‘Wild Pollinator Package’ of agri‐environment measures.

It has recently been argued that narratives around salient science are crucial to generating evidence‐informed action in the policy arena (Dudley, 2013; Rose, 2015). Put very simply, the narrative here goes something like this: ‘Some pollinators are declining and we depend on them for food production, so it is in our interest to stop these declines. If species are declining, it is either because they lack specific resources, or because one or more risk factors are reducing their numbers faster than they can reproduce. Some risks to pollinators, especially pesticides and climate change, are difficult to quantify and politically challenging to manage. An alternative is to focus policy on resources that are lacking. The role of pollinators in food production largely takes place on farmland and it has been demonstrated for birds that the best way to reverse species declines on farmland is by generating a simple, generic, flexible package of management options that are easy for farmers to implement. Farmers can be supported to take up the package with a combination of financial incentives, through agri‐environment schemes and advice. If it is possible to produce such a package for wild pollinators, then it has a good chance of benefitting pollinators.’

While many may not agree with all elements of this narrative, it is compelling because it combines an issue of strong public concern with emerging scientific evidence, and, most importantly, it culminates in an apparently sensible, practical solution.

In this paper, we show how it was possible to propose a package of agri‐environment measures for wild pollinators based on their ecological requirements, using the best available evidence and clearly accounting for the uncertainties. The approach was developed specifically to inform the design of the Wild Pollinator and Farmland Wildlife Package (described briefly in Table 1), which now forms part of the new Countryside Stewardship scheme (Defra, 2014b), and is a key element of the National Pollinator Strategy (Defra, 2014a). Its initial outcomes fed directly into the process of designing the pollinator elements of the package, although they were not the only influence on this policy formulation process. The Wild Pollinator and Farmland Wildlife Package was designed by Natural England, with input from a wide range of stakeholders, including farming organisations, academics, and conservation groups focused on a variety of taxa (not just pollinators, but also birds, plants, bats, and other invertebrates).

**Table 1:** Summary of the basic (mid‐tier) Wild Pollinator and Farmland Wildlife Package

Resources	Select one or more of the following Countryside Stewardship options	Minimum per 100 ha of farmed land	Maximum per 100 ha of farmed land
**Nectar and pollen sources for insect pollinators and insect‐rich foraging for birds** (OBLIGATORY)	**Arable or mixed farms**	1 ha in total	3 ha in total
	AB1 Nectar flower mix		
	AB8 Flower‐rich margins and plots		
	AB15 Two‐year sown legume fallow		
	AB16 Autumn sown Bumblebird mix		
	AB11 Cultivated areas for arable plants (*no more than 25% of the resource area*)		
	**Pastoral or mixed farms**	2 ha	4 ha
	GS4 Legume and herb‐rich swards (or OP4 Multi‐species ley)		
	GS2 Permanent grassland with very low inputs		
	**Mixed farms only:** GS17 Lenient grazing supplement		
**Winter food for seed‐eating birds (**OBLIGATORY for arable and mixed farms, OPTIONAL for pastoral farms)	AB9 Winter Bird Food (or OP2 Wild bird seed mixture)	2 ha	3 ha
	Can also select up to **7.5 ha** per 100 ha of AB6 Enhanced Overwinter Stubble or up to **15 ha** per 100 ha of AB2 Basic Overwinter Stubble (or OP1 Overwintered stubble)		
	**Pastoral or mixed farms**		
	GS3 Ryegrass seed‐set as winter/spring food for birds		
**Hedgerows** (OPTIONAL for arable and mixed farms, OBLIGATORY for pastoral farms)	BE3 Management of hedgerows	500 m	2000 m
**Nesting habitat for insect pollinators and birds** (OBLIGATORY for pastoral farms)	GS1 Take field corners out of management	0.5 ha	2 ha
**In‐field breeding habitats for skylarks** (OPTIONAL for arable farms)	AB4 Skylark plots	2 per ha of winter wheat	2 per ha of winter wheat
**Variable grassland sward structure to provide insect‐rich foraging for birds (**OPTIONAL for pastoral farms)	GS17 Lenient grazing supplement	1 ha	4 ha
**Ponds and ditches** (OPTIONAL)	WT1 Buffering in‐field ponds and ditches in improved grassland	As required	As required
	WT2 Buffering in‐field ponds and ditches on arable land		

This table shows the basic features of a package or bundle of agri‐environment measures that will be incentivised if adopted together under the new Countryside Stewardship scheme in England. The package is slightly different for arable, mixed, and pastoral farms as indicated. Details of scoring and how funding allocations will be decided were not apparent at the time of writing. A higher tier package also exists, with higher provision of pollinator resources, a specific requirement to provide floral resources on 0.5 ha in spring and autumn, and further optional additions including traditional orchard management and nest boxes for bees. Source: Natural England.

The approach and the calculations presented here are necessarily crude and rely on many assumptions and shortcuts. The basic structure of the Wild Pollinator and Farmland Wildlife Package has now been decided, although some of the best data that could have informed its design are still not available in the public domain, and there are several ongoing strands of research that could feed into it. However, as pointed out above, policy windows are short‐lived and decisions must be made, informed by the best available evidence. This paper documents the evidence that was available at the time. There was consistent agreement among those designing the Package that it needed to be flexible, and open to change as new knowledge became available.

## Method and approach

To propose a package of agri‐environment measures to support or reverse declines in wild pollinators based on their ecological resource requirements, four key questions must be answered:

1. Which pollinator species should be the target of the Wild Pollinator Package?
2. Which resources are currently limiting populations of these species in English farmed landscapes?
3. Which management options that could be supported through agri‐environment schemes provide these resources?
4. What area of each option is needed to provide sufficient resources to support populations of (or reverse declines in) the focal species?

In the remainder of this paper, we take each question in turn and outline how it is possible to provide answers to some elements of these questions for wild bees, using a combination of published and new ecological information. What follows must be considered through the lens of the underlying narrative that opened this particular policy window. For each of the questions, we have developed scientific responses that keep the focus on pollinators for food production and respect the need for simple but effective solutions.

### Which pollinator species should be the target of the English, wild pollinator package?

There are hundreds of species of wild pollinator in England. The term can be interpreted to include any flower‐feeding insect, thus including many species of Lepidoptera, aculeate Hymenoptera and Diptera, and some species of Coleoptera and Hemiptera. It is not easy to imagine building a simple package of measures expected to benefit all these species. Even if you focus only on bees, there are more than 250 wild species in the U.K. However, to develop quantitative answers, particularly to the final question – how much of each option is needed? – it is necessary to take a species‐based approach, or at least to define a generic set of species that can be expected to represent broadly the demands of a more diverse set.

We have focused only on wild bees, rather than all wild pollinators, for three main reasons. First, the ecological characteristics of bees make it feasible to answer these questions crudely, but relatively quickly. They are central place foragers, feeding exclusively on nectar and pollen as larvae and adults, and requiring these resources within foraging distance of their nest site. Other insect groups such as flies and butterflies have more varied feeding requirements and, for flies at least, there is very limited knowledge about how they move through landscapes. Second, it is often stated that bees are the most important insect pollinators (Klein *et al*., 2007; Roulston & Goodell, 2011). Clearly some U.K. plant species have floral forms adapted to pollination by other insect groups, such as moths or flies, but these are in the minority and bees are often important secondary or even primary pollinators for them (Rosas‐Guerrero *et al*., 2014). The implications of the focus on wild bees, and on a very limited subset of species, are considered in the discussion. Third, there is very strong evidence of declines in bee diversity in the UK during the period of agricultural intensification in the early to middle 20th century (Carvalheiro *et al*., 2013; Ollerton *et al*., 2014).

One possible approach similar to the Farmland Bird Package is to identify a set of wild pollinator species associated with farmland, but known to be declining. In summer 2014, a working group was set up by Natural England to identify such a set of species. Using a combination of expert judgement, and data from an ongoing Red Listing exercise led by the Bees Wasps and Ants Recording Society, they initially identified the species listed in Table S1 of File S1. As shown in Table S1 of File S1, half (six) of the selected declining bee species are moderately specialised in their choice of pollen sources (choosing yellow Asteraceae, Fabaceae or a few species in the Dipsacaceae). Using these bee species to assess resource requirements for wild pollinators more broadly would risk giving too much weight to these few plant families, so these species were not used to assess the resource requirements in the remainder of this paper. Nonetheless, identifying them gives a good indication of the specific resources likely to be associated with declining bees, which advisors can build into seed mixes or management on holdings in areas that contain these species.

An alternative approach is to focus on species actually delivering a pollination service to English crops, for food production. Kleijn *et al*. (2013) documented wild bee species frequently recorded as visitors to crop flowers (making up at least 5% of the visitor community) from 42 studies across Europe. Their list contains just 41 bee species. From this list, we selected six species known to exist in England that were very commonly recorded visiting flowers of crops grown in England. These species, and the criteria for their selection are shown in Table 2. Other species of bee that are common, generalised species in England, such as for example, *Bombus hortorum* (Linnaeus), are not included because they were only recorded in a limited number of datasets by Kleijn *et al*. (2013).

**Table 2:** Six species of wild bee most frequently recorded as dominant pollinators (>5% of recorded visits) for studies of flowering crops grown in the U.K

Latin name	Common name	Activity period	Reason for selection
*Bombus terrestris* (Linnaeus, 1758)*	Buff‐tailed bumblebee	March–October	Identified as a dominant crop flower visitor in more than 75% of studies and on all crops studied of those likely to be grown in the UK.
*Bombus lapidarius* (Linnaeus, 1758)	Red‐tailed bumblebee	March–September	
*Bombus pascuorum* (Scopoli, 1763)	Common carder bumblebee	March–October	Identified as a dominant crop flower visitor in multiple studies, on at least four crops.
*Andrena flavipes* Panzer, 1799	Yellow legged mining bee	March–October	
*Andrena haemorrhoa* (Fabricius, 1781)	Early mining bee	March–July	
*Andrena cineraria* (Linnaeus, 1758)	Ashy mining bee	March–July	Often recorded in France and Germany as a dominant bee visitor to oilseed rape flowers (>5% of visitor abundance in seven different studies). This bee is expanding its range in the UK.

* In Kleijn *et al*. (2013) this species is combined with the *Bombus lucorum* sensu lato species complex, as workers are indistinguishable in the field.

Flower preferences for these species are shown in Table S3 of File S1. All six species are polylectic. Sources: Reasons for selection: Kleijn *et al*. (2013). Phenology information: see Table S8 of File S1.

Of course, a focus on a few currently abundant and widespread species – those likely to be delivering the pollination service to crops – may not provide appropriate resources for species that are actually declining. This question will be returned to in the discussion.

### Which resources are currently limiting populations of these species in English farmed landscapes?

Three main factors have been suggested to regulate bee populations directly: food resources, nesting resources, and the availability of refugia from incidental risks, including pesticide exposure (Roulston & Goodell, 2011). For wild bees, Roulston and Goodell (2011) looked for evidence that each of these actually limits numbers of bees. They found strong evidence for food resources limiting bee populations, but not for the other two factors. The loss of floral resources in agricultural landscapes is a prominent and much‐discussed driver of decline, often argued to be the most important (for example, Carvell *et al*., 2006; Carvalheiro *et al*., 2013; Ollerton *et al*., 2014). It includes declines in particular types of flower for bumblebees for example (Carvell *et al*., 2006) and large‐scale loss of flower‐rich habitats such as species‐rich grasslands.

In contrast, Vanbergen *et al*. (2013) reviewed the state of knowledge on pressures driving pollinator declines and concluded that multiple pressures are responsible, including examples leading to all three of the direct regulatory effects above. For this reason, in identifying options that deliver resources (question 3), all three potential limiting factors were considered.

Once a resource has been identified as potentially limiting, whether it is actually limiting in a particular landscape depends on (i) the critical quantity of the resource required for the population to stabilise or increase and (ii) the level of resource currently available in that landscape.

In birds, population dynamic modelling parameterised by real population data was available to show critical levels of key population parameters that had to be reached in order to reverse declines (Vickery *et al*., 2008). Resources were identified that could be linked to the key parameters. For example, if the key parameter was first‐year survival, winter food was the critical resource assumed to be limiting, whereas if the key parameter was young/nest, summer food was assumed limiting. Threshold quantities of specific habitats assumed to provide these resources were estimated, using evidence available at the time linking habitat management to bird demographics (the key papers were: Aebischer & Ewald, 2004; Morris *et al*., 2004; Gillings *et al*., 2005). There was no attempt to calculate how much of each specific resource the birds actually need to sustain their densities or increase the key parameters, or to calculate how much of each resource the suggested management options provide.

For wild bees, there were at the time no parameterised population models to identify key population parameters, so the same process could not be replicated. For honey bees (Becher *et al*., 2014) and solitary bees (Everaars & Dormann, 2014), models have recently been built that simulate colony dynamics or nest‐stocking behaviour and include interactions with external influences such as landscape‐scale forage provision or pesticide exposure. So far, neither of these models has been parameterised with real population or community data or used to identify key limiting factors in farmed landscapes.

However, there was considerable information available about the demand for floral resources by bees and the provision of floral resources in agri‐environment options, elements not considered for the Farmland Bird Package. This information has been used to address the question of resource limitation in this study.

There was no quantified information about demand for, or supply of nesting resources or refugia. However, as yet there is very little evidence that nesting resources and refugia are limiting populations or can lead to an increase in wild bee numbers if enhanced in farmland. So although some options are included in the package to provide these resources, the quantification of the amount of habitat required (question 4) was based on floral resources only.

Quantifications of existing nectar provision for wild pollinators at a landscape scale or higher (up to national maps) are currently being developed for the U.K., based on average levels for each habitat and land cover maps (M. Baude *et al*. unpublished). Similar, less detailed data were available through the Countryside Survey on densities of bee forage plants (not flower numbers or nectar provision) in different habitat types (Smart *et al*., 2010). Information about the availability of both floral and nesting resources at a national level could also be drawn from expert judgement, using the approach for estimating wild bee abundance in the ‘Lonsdorf’ model (Lonsdorf *et al*., 2009). This has been widely used for mapping potential pollination services and their value (for example, Bai *et al*., 2011; Maes *et al*., 2012). However, at the time of policy development, Lonsdorf indices for English habitats were not available.

In theory, such landscape or national‐scale information could be used to target options providing floral resources to areas where these resources are deficient. However, one of the main requirements for the agri‐environment package is that it must be very simple for farmers and applicable in a generic form across the country. Targeting by existing resource provision would entail the package itself being tailored to suit different landscapes. Ecologically, this would be extremely sensible, but practically, it was not.

### Which management options that could be supported through agri‐environment schemes provide these resources?

Specific options within the existing English agri‐environment scheme (Entry Level Stewardship) that support pollinators have been identified in two separate exercises led by stakeholders – the Campaign for the Farmed Environment and Natural England. These are shown in Table 3, as the CFE and ELS Handbook, respectively. An evidence‐based prioritisation process (Sutherland *et al*., 2011; Sutherland *et al*., 2012) identified 54 possible interventions that could benefit wild bee populations in the UK, and scored each for importance and certainty of evidence, based on evidence compiled by Dicks *et al*. (2010). Table S2 of File S1 lists these interventions, ranked according to an Advocacy Priority Index, which selects interventions of high importance and high certainty. The top ranking interventions applicable on farmland (excluding those too general to be relevant, such as ‘introduce agri‐environment schemes generally’) are also indicated in Table 3.

**Table 3:** Management options that supply resources for pollinators

Option	Identified by					Pollinator resources supplied
	CFE	ELS Handbook	Evidence‐based prioritisation	Lonsdorf index	Countryside Survey	
Nectar flower mixture	√	√	√			Food
Hedgerow management for landscape and wildlife		√			√	Food, nesting, refugia
Combined hedge and ditch management		√			√	Food, nesting, refugia
Management of woodland edges		√		√	√	Food, nesting, refugia
Supplement to add wildflowers to field corners and buffer strips on cultivated land	√	√	√		√	Food
Management of field corners	√	√				Food, nesting, refugia
Permanent grassland with very low inputs	√	√				Food, nesting, refugia
Ryegrass seed‐set as winter/spring food for birds		√				—
Legume‐ and herb‐rich swards	√	√			√	Food
Unsprayed and/or unfertilised cereal headlands	√					Food, refugia
Selective use of spring herbicides	√					Food, refugia
Create patches of bare ground for ground‐nesting bees			√			Nesting
Restore species‐rich grassland vegetation			√	√	√	Food, nesting, refugia
Restore lowland heathland			√	√		Food, nesting, refugia
Provide set‐aside areas in farmland			√			Food, nesting, refugia
Provide artificial nest sites for solitary bees			√			Nesting
Leave arable field margins uncropped with natural regeneration			√		√	Food, nesting, refugia

CFE = identified as a voluntary measure for pollinators by the Campaign for the Farmed Environment (Campaign for the Farmed Environment, 2013). ELS Handbook = identified in the Entry Level Stewardship Handbook (ELS) as a priority option when managing land for butterflies, bees and grassland (Natural England, 2013). Evidence‐based prioritisation = one of the top 10 applicable on farmland, from a ranking of interventions based on importance for conservation and evidence synthesis (Dicks *et al*., 2010; Sutherland *et al*., 2011, 2012; Table S1, File S1). Lonsdorf index = management option linked to a habitat type identified with high floral resource or nesting suitability by Kennedy *et al*. (2013). Countryside Survey = management option linked to a habitat type identified with high mean numbers of nectar plants per plot by Smart *et al*. (2010). The resources for pollinators are discussed in the main text (question 2: Which resources are currently limiting?).

Assessments of floral resource provision and/or nesting suitability for different farmland habitats are available from two sources. One is a study of landscape effects on bee abundance and diversity, conducted across 39 studies globally (Kennedy *et al*., 2013). Bee (especially social bee) abundance and species richness in the study landscapes could be predicted using the ‘Lonsdorf Landscape Index’, calculated for surrounding landscapes using indices for floral and nesting resource. In this study, the habitat types with the highest floral resources (mean floral resource index >0.6, judged by individual study authors, not measured) were: semi‐natural grassland, shrubland (scattered woody vegetation, <6 m tall, not touching or interlocking – does not include hedgerows), and orchards/vineyards. The most suitable habitats for nesting (mean nesting suitability index >0.6) were semi‐natural grassland, shrubland, broadleaved forest, mixed forest, and woody wetland. The other source is UK Countryside Survey data on the densities of bee forage plants in different U.K. habitat types. Smart *et al*. (2010) identified calcareous grassland, rivers and streams, boundary and linear features, neutral grassland, and broadleaved woodland as the broad habitats with the highest mean numbers of nectar plants per plot, although their use of plant, rather than flower density data means they would be likely to underestimate the value to bees of heathlands dominated by flower‐rich ericaceous shrubs. Management options that create or maintain these resource‐providing habitat types are indicated in Table 3.

Almost all the management options identified by more than one of the sources listed in Table 3 are included in the basic Wild Pollinator and Farm Wildlife Package (Table 1). The exceptions are restoration of species‐rich grassland and lowland heath, and management of woodland edges. Woodland edge management is included in the higher tier version of the Wild Pollinator and Farm Wildlife Package, for the provision of spring flowers. Species‐rich grassland and heathland are targeted in their own right as part of the Countryside Stewardship scheme, which has management and restoration of non‐farmed habitats as a key objective. The basic (mid‐tier) Wild Pollinator and Farm Wildlife Package discussed here has been developed to compliment this habitat‐focused approach in more intensively managed areas of farmland within the wider countryside.

### What area of each option is needed to provide sufficient resources to support populations of (or reverse declines in) the focal species?

To answer this question, it is necessary to know (i) how much of the limiting resources are needed to support viable populations, or reverse population declines and (ii) how much of these resources are supplied per hectare of selected agri‐environment options. As there is substantial uncertainty in both areas, largely as a result of a paucity of relevant scientific data, we aim only to provide a range of possible values.

As discussed above, we focus on floral resources and the common, generalist, service‐providing species in Table 2. According to Muller *et al*. (2006) and Everaars and Dormann (2014), we focus only on pollen, as this is a key resource for reproduction in bees and there are data available for rates of pollen consumption by bee larvae.

Equation 1 is a simplistic way to calculate pollen demand/area over time:

(1) PD=PB×BN×N

where *PD* is the total pollen demand (mm^3^) per 100 ha per month, *PB* is the pollen consumed by individual bee larvae (mm^3^), *BN* is the number of new individual bees per month raised in a single nest (including workers and reproductive social bees), and *N* is the density of individual colonies or nests per 100 ha. This is clearly over‐simplified, as it ignores colony dynamics (interactions between numbers of workers and reproductives for social bees), and differential foraging ranges [many bee species forage beyond a single 100 ha block and foraging ranges are plastic, depending upon provision of floral resources in the landscape (Carvell *et al*., 2012)]. However, the value of such a simple equation is that it allows a pollen demand per 100 ha to be calculated from parameters that are known or can be estimated, for some species at least. The process we followed to do this is explained in detail in the Materials and methods of File S1.

(2) $$F=\frac{PD}{PF}$$

Equation 2 calculates flower demands from pollen demands, where *F* is the number of floral units per 100 ha per month, *PD* is the total pollen demand (mm^3^) per 100 ha per month from eqn 1, and *PF* is the average pollen volume (mm^3^) per floral unit. A floral unit can be a single flower or an inflorescence for species with tightly clustered flowers, such as those in the family Asteraceae or Dipsacaceae. Two sources of data exist on the volumes of pollen produced per floral unit of particular plant species. In the published literature, Muller *et al*. (2006) reported pollen volumes for the 16 plant species on which their specialised solitary bees were feeding. More recently, a dataset of pollen volumes per flower for 153 native UK plant species has been developed (see Materials and methods, File S1). Both datasets are summarised in Table S6 of File S1. For the purposes of crude calculation, here we have used an average value of pollen per floral unit from these datasets (see Table 5, assumption 5, for discussion of the implications of this).

**Figure 1:**
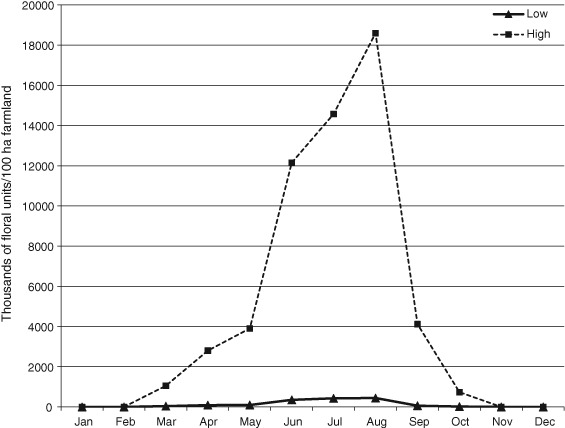
Combined demand of pollen for larval rearing, in floral units per 100 ha, for six dominant crop‐pollinating wild bee species. Calculations are based on eqns 1 and 2, using parameter values shown in Table S7 of File S1, and phenologies given in Table S8 of File S1. The species used are shown in Table 2. Upper and lower bounds are given to show the range of uncertainty.

**Figure 2:**
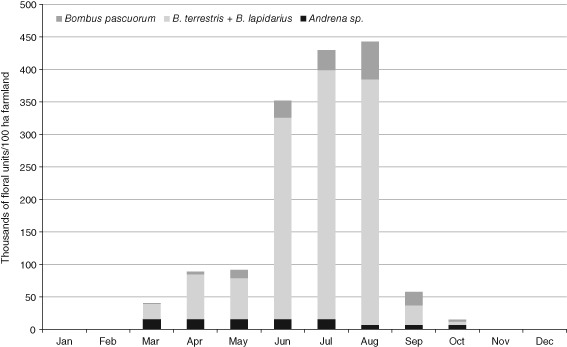
Low estimates of pollen demand for larval rearing, in floral units per 100 ha, for the six wild bee species, broken into crude functional groups. Bombus pascuorum is a long‐tongued bumblebee, B. terrestris and B. lapidarius are short‐tongued bumblebees, and Andrena sp. represents three solitary species: Andrena flavipes, Andrena haemorrhoa, and Andrena cineraria. Andrena flavipes has two generations per year, and is the only Andrena species of the three with floral demands in August, September, and October. Calculations are based on eqns 1 and 2, using parameter values shown in Table S7 of File S1, and phenologies given in Table S8 of File S1.

To provide an indication of the level of uncertainty in these calculations, we calculated upper and lower bounds for the number of total floral units, using high and low estimates for pollen demand per larva, bumblebee colony density, and pollen volume per floral unit (shown in Table S7, File S1). The results are shown in Figs 1 and 2 (broken down into types of bee for the low estimates only). An alternative approach would be to calculate estimated standard errors (SEs) or coefficients of variation for the expected floral resource demand, based on variances of individual variables in eqn 2 (for example using equations originally described by Goodman, 1960). At this stage, with so many assumptions and unknowns, we felt this would suggest a misleading level of accuracy and be less informative than clearly providing high and low estimates.

Figure 1 illustrates the very large range of uncertainty in how many floral units are actually needed by these bee species. In the summer months, when the bumblebee numbers are at their highest, the number of ‘average’ floral units demanded by just six common bee species per month per 100 ha is somewhere between 350 000 and 18.6 million. Figure 2 illustrates how this flower demand breaks down among the types of bee, for the lower estimates only. It shows a strong dominance of bumblebees. The upper bound of calculated flower demand is even more dominated by bumblebees, with the greatest increase in demand from the long‐tongued species *Bombus pascuorum* (Scopoli) in the summer months, because of the wide range in estimates of colony density for this species (8 colonies per 100 ha in the lower set, 193 colonies per 100 ha in the upper set; see Table S5, File S1).

It is impossible to say whether adding these quantities of floral resource to existing agricultural landscapes would reverse the fortunes of declining wild pollinators or maintain populations of widespread common wild bees. One main reason is that we do not yet know where on Fig. 1 a line of actual floral resources in a real landscape would lie. The purpose of this exercise is to provide some ballpark figures, to show the possible range of requirements, for six widespread and common pollinator species.

#### How many flowers are provided per hectare by different agri‐environment scheme options?

For some of the management options listed in Table 3, average total flower densities in May, June, July, and August have been reported by Carvell *et al*. (2007, 2011). These are summarised in Table 4. Using the lower estimated floral demands for the focal bee species from Table S9 of File S1 (and Fig. 1), the maximum number of floral units needed per month for these months is 442 658 in August. From the results for all flowering species in Table 4 (Carvell *et al*., 2007), 2 ha of nectar flower mix can be expected to provide approximately 430 000 floral units per month between May and August, and wildflower mixture, 490 000 floral units per month. As actual floral unit densities were much higher in July and August than in May and June (see note to Table 4), we can infer that 2 ha of these options per 100 ha, or 2% of the farmed landscape, would be sufficient to supply the calculated pollen demands. In a later study, Carvell *et al*. (2011) measured much higher densities of bumblebee flowers in nectar and pollen mixture (420 000 flower per ha; Table 4), implying that 1 ha of good quality nectar flower mix could potentially supply sufficient pollen resources. Floral resources provided by these agri‐environment options are known to vary and were recently shown to increase with greater farmer experience and motivation to support wildlife (McCracken *et al*., 2015). In the Carvell *et al*., 2011 study, high flower density was probably a result of both improved composition of the seed mixtures and advice to farmers leading to better establishment and persistence of sown habitats.

**Table 4:** Numbers of flowers supplied by selected agri‐environment options on arable farmland, based on densities per m^2^ of floral units, scaled × 10 000 to get per ha values

Option and entry level stewardship code	Source	Mean density of floral units per ha per month*	Period of flowering time
Nectar flower mixture EF4	Carvell *et al*. (2007)*	215 342 (14 160)	May–August†
Nectar flower mixture EF4	Carvell *et al*. (2011)‡	419 970 (42 606)	May–August
Wildflower mixture EE3/HE10	Carvell *et al*. (2007)	244 841 (20 533)	May–August
Tussocky grass mixture EE3	Carvell *et al*. (2007)	19 525 (2 642)	May–August
Uncropped natural regeneration EF11	Carvell *et al*. (2007)	73 124 (7 463)	May–August
Cropped cereal headland EF9	Carvell *et al*. (2007)	34 916 (5 575)	May–August

* Adapted from data on 6 m field margins, averaged over 3 years (2002–2004), 92–108 plots per year. Mean numbers of floral units of all species in flower at any sampling time.

† Flower densities were much higher in July and August than May and June, particularly for pollen & nectar mix, but reported values under each option are averages per month (Carvell *et al*., 2007).

‡ Adapted from data for nectar flower mix planted in blocks of between 0.25 and 1 ha, averaged over 3 years (2005–2007), 100–118 plots per year. Mean numbers of floral units of bumblebee‐visited plant species in flower at any sampling time are presented.

Standard errors (in brackets) represent variation between sites, months, and years.

Flower densities on these options in September and October could be reduced to almost nothing by mowing as prescribed under the Schemes (Carvell *et al*., 2007; Tarrant *et al*., 2013), in which case bees would need to rely more on later‐flowering plant species elsewhere in the landscape, such as *Hedera helix* L. (Garbuzov & Ratnieks, 2014), or unmown areas left as refugia (Kühne *et al*., 2015).

The sown flower agri‐environment options seldom supply flowers in March and April, crucial months for bumblebees, which are founding colonies, and for many solitary bee species, for which these months are key parts of the flight period. Spring flowers can be provided by flowering hedgerows. As an illustration of the kind of calculations that will be possible when bee diets and flower densities are better characterised, Table S10 of File S1 derives estimates of the length of single species hedgerow that would be needed to supply pollen demands for larval rearing of the six focal bee species during the spring months, for two hedgerow species *Prunus spinosa* L. and *Crataegus monogyna* Jacq. These species tend to flower in March/April and May. respectively. The calculations are based on eqn 3, in which *H* is the length of hedgerow required per 100 ha per month (m), *PD* is the total pollen demand (mm^3^) per 100 ha per month, *PHF* is the average pollen per floral unit of the hedgerow species (mm^3^), and *FM* is the number of floral units per metre of hedge. For the low estimates of pollen demand, the upper end of these estimates is around 765 m of *P. spinosa* hedge to supply the April pollen demand. The lengths demanded are highest for *P. spinosa* because both flower densities and pollen volume per floral unit are lower than those for *C. monogyna*. Of course, hedgerows are seldom made up of single species, and pollen demands in April may also be met by early flowering herbaceous species such as comfrey *Symphytum officinale* L., white deadnettle *Lamium album* L., and dandelion *Taraxacum officinale* F.H. Wigg.

(3) $$H=\frac{PD}{PHF\;\times FM}$$

In the Wild Pollinator and Farmland Wildlife Package for pastoral farms (Table 1), legume and herb‐rich swards are a key option for nectar and pollen provision. This requires grass swards with 10% *Trifolium pratense* L. cover (largely visited by long‐tongued bees) and 10% other herbs, including legumes. Using Equation S1 of File S1, and measurements of the floral density and pollen volume per floral unit for *T. pratense*, we can calculate that 7–8 ha of legume and herb‐rich sward would be needed per 100 ha to supply the pollen demands of *B. pascuorum* in June and July, if it was feeding entirely on *T. pratense* (Table S11, File S1). This exceeds the maximum 4 ha required in the Package.

At the other end of the scale, using the high end estimates of flower demand from Table S9 of File S1 and the flower densities from Table 4, Tables S10 and S11 of File S1, these six bee species would need 44 ha in every 100 ha, or 44% of the farmed landscape sown as well‐managed nectar flower mix EF4 (the total flower demand for August, 18.6 million floral units, requires 44.3 ha containing 419 970 floral units each; data from Carvell *et al*., 2011) and 13.8 km of flowering hedge per 100 ha (Table S10, File S1), to meet the April pollen demand for their larvae. The long‐tongued bumblebee species, *B. pascuorum* would require well over 100 ha of legume and herb‐rich sward per 100 ha (Table S11, File S1) to supply its July pollen demand solely from red clover.

#### Layout of resources in the landscape

There was a preference from policymakers and stakeholders to build a package for a single 100 ha block (1 km^2^) of farmland, because it is straightforward and easy to understand, and has precedent in the Farmland Bird Package. As bees are central place foragers, they repeatedly fly between nesting sites and floral resources to feed their larvae. It is, therefore, important to consider the spatial limitations on where resources can be placed.

Table S12 of File S1 provides estimates of maximum and mean foraging ranges for the focal bee species, either calculated from intertegular distance (distance between wing bases) according to an equation derived from empirical observations by Greenleaf *et al*. (2007), or empirically measured by Carvell *et al*. (2014). These maximum foraging range estimates for bumblebees are higher than other authors have estimated. For example, Darvill *et al*. (2004) derive estimates of around 300 and 600 m for *B. pascuorum* and *B. terrestris*, respectively, in a complex and diverse landscape. We know foraging range is flexible and dependent on the resources provided in the habitat (Carvell *et al*., 2012). However, even the lower estimates of foraging range allow bumblebees from a single colony to cover most of the area with a 100 ha block (1 km^2^).

Several recent studies imply that the configuration of landscape features (the way they are arranged in the landscape) has only a weak or no effect on bee populations or population persistence (Franzen & Nilsson, 2010; Kennedy *et al*., 2013; Everaars & Dormann, 2014). Although there is both experimental and modelling evidence that linear features linking patches of floral resource promote movement of bees and other pollinators through landscapes (Cranmer *et al*., 2012; Hodgson *et al*., 2012), and can enhance pollen transfer between plants in those patches (Townsend & Levey, 2005; Cranmer *et al*., 2012), this does not mean that linking patches of resource or habitat is important to support bee populations.

Given that both bumblebees and larger solitary bees have estimated maximum foraging ranges of close to or greater than 1 km, and the lack of evidence that spatial configuration is important, it seems that supplying the appropriate number of flowers somewhere within each 100 ha block should be sufficient. This may not be the case for smaller species of solitary bee, such as members of the genera *Halictus* and *Lasioglossum*¸ not considered here.

## Discussion

From evidence available at the time when the policy opportunity arose, it was possible to draw tentative upper and lower quantitative bounds on the demand for floral resources by a set of common, widespread, pollinating bee species. As noted by Muller *et al*. (2006), these flower requirements are surprisingly high. They are calculated for only six bee species, at densities estimated from real farmed landscapes. Flower‐visiting insect communities that have been documented always have substantially more species than this. For example, the five English plant–insect visitor network datasets reported in Carvalheiro *et al*. (2014) include between 16 and 243 flower‐visiting species that are potentially pollinators.

When compared against floral resources likely to be provided by the key agri‐environment options included as nectar and pollen sources in the Wild Pollinator and Farmland Wildlife Package, it was shown that elements of the Package for arable/mixed farms are sufficient to supply a basic set of pollinating bee species with enough pollen to feed their larvae at the lowest estimates only, taking the minimum values for estimated parameters where a range was available and assuming the bees are either polylectic or will consume pollen from one or more of the flowering species provided. In the case of legume and herb‐rich sward supplying pollen for long‐tongued bumblebees on pastoral farms, the Package as currently prescribed may benefit from increasing the coverage of red clover in the legume and herb‐rich swards from 10% to at least 20%.

Our estimated requirement for 2% flower‐rich habitat within 100 ha of farmland falls within the range generated by Carvell *et al*.'s study of bumblebee colonies across an enhanced agricultural landscape. That study suggests that landscapes supporting between 1% and 3% cover of suitable flowers should allow bumblebee workers of most species to forage at or below their species mean distance from the colony (Carvell *et al*., 2014), assuming that by reducing the cost of foraging and thus net energy expenditure, colony survival is likely to be enhanced.

The high‐end estimates of pollen demand in this paper generate habitat quantities that are way out of reach for agri‐environment (or any other current) policies.

Responding to policy needs with relevant ecological evidence almost always means working with incomplete knowledge and large uncertainty. Assumptions have to be made, based on the best available evidence combined with expert judgement. Ideally, the best available evidence should be derived from  hierarchical synthesis of evidence (Dicks *et al*., 2014a) and rigorous processes for compiling expert judgement (Dicks *et al*., 2014b), with proper characterisation of the sources of uncertainty, but these processes are not always possible with limited resources. At the very least, it is important to be explicit about the assumptions, the uncertainty they introduce and the implications of these uncertainties. This is important because, in many cases where policy draws on scientific evidence, including in this case, the underlying assumptions and uncertainties are not described as part of the resulting policy.

The main assumptions that were made in quantifying how much flower‐rich habitat is needed to support pollinators in English farmed landscapes are described in Table 5. An overview of the level of accuracy and sources of uncertainty in the parameter estimates used for Eqns 1, 2, 3 and Equation S1 of File S1 is provided in Table S13 of File S1.

**Table 5:** Six assumptions underlying the calculations of required area of flower‐rich habitat, and their implications for decision‐making

Assumption	Implications
(1) Requirements for all pollinators can be quantified on the basis of a small set of bee species	Resources important for non‐bee pollinator taxa are overlooked. The most prominent are larval food plants for Lepidoptera, and larval resources for a diversity of Syrphidae, including aphid prey for aphidophagous species and freshwater for detritivorous species. These groups, and specialist bee species relying on specific habitats or plant species, may not be supported.
(2) Floral resources are limiting for bees. Other resources, such as nesting resources and refuge from incidental risks are not limiting.	If nest sites or refuge from risks are the limiting factor, providing additional floral resources will not stabilise or maintain pollinator populations.
(3) Bee densities recorded in existing studies represent a viable density for provision of pollination service or reversal of declining populations	Densities of bees and other pollinators in real landscapes must be higher than the single species densities measured in the studies cited here, because of the diversity of other flower‐visiting insect species using the same resources in real communities. The bumblebee colony densities are likely minimum estimates, being based on the number of related sister workers sampled during a short phase of the colony cycle. The solitary bee nest densities are speculative, based on measured densities of two less common bee species in Sweden (see Materials and methods of File S1). It is therefore not possible to say whether providing this quantity of resource is enough to provide the pollination service or reverse declines.
(4) All pollen in flower heads is available to bees	If only a proportion *P* of pollen is actually used by bees, then flower requirements would be higher by a factor of 1/*P*. Muller *et al*. (2006) estimated, based on measurements of five plant species, that 40% of the pollen held in a flower is available to foraging bees. If correct, then the flower requirements would all need to be multiplied by a factor of 2.5. However, other studies have documented 95.5 or 100% of pollen being removed from *Cucurbita pepo* or *Campanula rapunculus* flowers respectively, by foraging bees (Willis & Kevan, 1995; Schlindwein *et al*., 2005).
(5) All flowers are equally distributed in habitats	We calculated overall flower demand based on an average pollen volume per floral unit, measured across a whole set of flowering plant species, with no consideration of the relative frequency of different flowers in actual landscapes, or the considerable variation in pollen production between plant species. The implications of this assumption require further analysis, adjusting the pollen provision according to relative abundances of different plant species found in farmed landscapes.
(6) Adding fixed levels of specific resources is a generic solution that will always help	Ecological research clearly shows that the attractiveness of flower strips for bees and other pollinators depends on the ‘ecological contrast’, or the relative density of floral resources in the immediate surroundings (Scheper *et al*., 2013). Adding a fixed amount of floral resource will not have the same effect everywhere. Such effort should be targeted towards areas where there is an intermediate degree of complexity in the surrounding landscape, providing a diverse community of pollinators to benefit from the additional resource, but also farmland that is relatively intensive, providing ecological contrast with the immediate surroundings.

Some caveats must accompany the calculations. The main limitations of the approach are: it is based on six common wild bee species that do not fully represent the suite of wild pollinators (Assumptions 1 and 3, Table 5); existing floral resources in the landscape are not taken into account (Assumption 6, Table 5); the pollen demands are only calculated for rearing larvae to adulthood, not for supplying adult bees once they are flying; no account is taken of other nutritional needs of bees, such as sugar, or specific amino acids, nor of variable nutritional quality of different pollens. Some pollen types do not support bumblebee larval development at all if the only source of food, as found for *Taraxacum* pollen by Genissel *et al*. (2002).

Straightforward calculations of products and dividends, such as those in eqns 1 and 2, are very sensitive to parameter levels. If the estimate of any parameter changes, the total flower demand for the affected bee species will change by an equivalent factor. When these values are summed across species and partitioned across months, as in Figs 1 and 2, the sensitivity of the overall model to different parameters deserves exploring. This will be the subject of future work.

The accuracies of our parameter estimates are indicated by standard errors or standard deviations in Table 4, Tables S3.1, S3.2, S4, S5, S6, and S10.1 of File S1. The largest sources of error are in the estimates of pollen volume per flower for individual plant species (Table S10.1, File S1). Everaars and Dormann (2014) tested the sensitivity of their individual‐based model of solitary bee foraging to changes in parameter values. The most relevant response variable to the question addressed here was the number of brood cells the *in silico* bees could produce in a day (strongly related to reproductive success). This response was most sensitive to the volume of pollen per flower (parameter elasticity 0.813 for soil‐nesting bees). It was not sensitive (parameter elasticity <0.05) to flower density (with values ranging from 10 to 100 floral units per m^2^), or to landscape variables such as fragmentation, landscape quality or foraging habitat availability. From this, it is clear that refining estimates of the volume of pollen per flower in plant species on which target bees are actually feeding is the highest priority for improving these estimates. This will involve linking empirically measured pollen volumes to actual foraging choices for each species.

Clearly six common wild bee species do not fully represent the suite of pollinators providing the pollination service on farms to crops and wildflowers. Although bees are identified as the dominant pollinators of crops, and are frequently recorded visiting crop flowers, pollinator communities for some crops, such as oilseed rape, are known to include other taxonomic groups such as flies (Garratt *et al*., 2014). Flower‐visiting insect communities are highly variable in space and time, and numbers of each species can fluctuate widely from year to year (for example, see Franzen & Nilsson, 2013). It is unlikely that the same group of pollinating insects provides the service consistently, in any place and across the country, and a number of studies have shown that this diversity is important to the delivery of pollination services (for example, Albrecht *et al*., 2007; Hoehn *et al*., 2008; Carvalheiro *et al*., 2010; Rogers *et al*., 2014).

Although specialist species are more likely to have declined in range (Biesmeijer *et al*., 2006; Carvalheiro *et al*., 2013), many U.K. species of bee and other pollinators are generalised, particularly in their choice of food resources, so it is quite easy to argue that supporting the widespread, common species will also support at least some of the declining, less common species. Evidence for this comes from the ability of generic agri‐environment options planted for pollinators to attract rare species from Table S1 of File S1, such as *Bombus ruderatus* and *B. muscorum* (Carvell *et al*., 2007).

While we do not have evidence that the common bee species from Table 2 have declined, it is also important to note that there is no evidence capable of revealing any ongoing trend in abundance of wild bee species. None of these common species show range declines, but that does not mean their numbers have not decreased over time in response to the same pressures as those facing the declining species. Declines in abundance inevitably come before range declines. Several species of farmland bird have declined in abundance by over 50% with little or no range decline since 1970 (Fuller *et al*., 1995; Gregory *et al*., 2004).

### A research agenda for pollinator conservation

This exercise exposes a huge amount of uncertainty affecting decisions about how to respond to pollinator declines. It might, therefore, be considered to represent a research agenda for pollinator conservation. The key areas of uncertainty it exposes are: the factors limiting wild bee populations, or causing changes to their populations and ranges; the target densities of pollinator species needed to reverse declines or supply pollination services; the nutritional requirements of wild pollinators; and the precise densities of different flowering species and quantities of nectar and pollen available in real agricultural landscapes. Active research is ongoing in all these areas, and answers can be expected at least for some species or communities in the coming years. Given the scale of uncertainty about the amounts of specific management options required to achieve the policy aim of supporting pollinators on farmland, the maximum and minimum area requirements for pollinator resources in the Wild Pollinator and Farm Wildlife Package may need to be revised as new evidence emerges.

## Supporting information


**File S1.** Supplementary Information.
