# Supplementary material for: How much flower‐rich habitat is enough for wild pollinators? Answering a key policy question with incomplete knowledge
Source: Ecol Entomol. 2015 Jul 2;40(Insects and Ecosystem Services 28th Symposium of the Royal Entomological Society of LondonS1):22–35. doi: 10.1111/een.12226 (PMC4737402; doi:10.1111/een.12226)
Supplement: Supplementary file 1 — File S1. Supplementary Information. [file EEN-40-22-s001.pdf]

SUPPLEMENTARY INFORMATION

How much flower-rich habitat is enough for wild pollinators? Answering a key policy question with incomplete knowledge

Authors: Lynn V. Dicks[1], Mathilde Baude[2], Stuart P.M. Roberts[3], James Phillips[4], Mike Green[5] and Claire Carvell[6]

[1] Department of Zoology, University of Cambridge, Downing Street, Cambridge, CB2 3EJ, UK.

[2] Collegium Sciences et Techniques, LBLGC EA 1207, Université d'Orléans, Orléans, France and School of Biological Sciences, University of Bristol, Bristol, UK.

[3] Centre for Agri-Environmental Research, School of Agriculture, Policy & Development, University of Reading, RG6 6AR, UK.

[4] Natural England, Riverside Chambers, Castle Street, Taunton, Somerset TA1 4AP, UK.

[5] Natural England, Mail Hub Block B, Whittington Road, Worcester, WR5 2LQ, UK.

[6] NERC Centre for Ecology and Hydrology, Maclean Building, Crowmarsh Gifford, Wallingford, Oxfordshire. OX10 8BB, UK.

Supplementary Materials and Methods

The text below describes specific approaches used to develop quantitative answers to question 4 in the main text: What area of each option is needed to provide sufficient resources to support populations of (or reverse declines in) the focal species?

Pollen demands (Equation1)

Pollen demand per bee

For solitary bees, Muller *et al*. (2006) provide an empirically derived regression equation that relates solitary bee dry body mass to pollen requirement per brood cell (per larva) for specialist species that feed on a few or just one plant species. The estimates of pollen required for 35 solitary bee species are translated into number of flowers or flower heads for the specific plant species involved, based on empirical measurements of pollen produced per flower. Muller *et al.* (2006) provide means and standard deviations for pollen volume per brood cell, body mass, and pollen volume per floral unit for the 35 bee or 16 plant species they consider.

We do not have dry body mass measures for the common crop flower visitor species listed in Table 2 (main text). Also, they are broad generalists, using pollen from a wide range of flower species rather than just one or a few flowering species. Even so, it is possible to get a range of estimated pollen volume requirements for bees in these size ranges, from the estimates of Muller *et al*. (2006) – these are given in Table S3.

Two studies in the literature provide data that can be used to estimate the mass (not volume) of pollen consumed by *Bombus terrestris* larvae in healthy laboratory colonies (Ribeiro et al. 1996, Genissel et al. 2002; Table S3.1). We found no estimates for quantity of pollen consumed by other bumblebee species, so we use these values for all bumblebee species.

Since pollen provision by flowers is measured by volume, not mass, we need to convert the bumblebee pollen demand into volume. The 'pollen' fed to captive bumblebees in these experiments almost certainly came from honey bee corbicular loads and is therefore mixed with syrup or nectar, and honeybee saliva to some extent. The density of these can be estimated from the mass of a honey bee corbicular load and its volume. Roman (2006) measured weights of individual pollen loads based on the number of loads and their overall weight, sampled in two years at two sampling times (early and late summer) from 19 *Apis mellifera* colonies in Poland (data in Table S3.2) . We could not find direct reports of the volume of corbicular loads, but Milne *et al.* (1986) reported corbicular area for two honey bee strains as close to 2mm^2 (1.909 ± 0.004 mm^2 for a high pollen-hoarding line and 1.874 ± 0.003 mm^2 for a low hoarding line). If we assume the corbicular load to be 2 to 3 mm deep and to fill the corbicular area, this gives a volume estimate between 4 and 6 mm^3. Using these estimates, we derive a range of density estimates from Roman's (2006) data (Table S3.2). Masses of corbicular pollen are converted to a range of volumes in Table S3.1 using the relationship *volume* = *mass*/*density*, to give a range of bumblebee pollen demand/ larva between 210 and 772 mm^3 per bumblebee larva.

Corbicular pollen may be denser than pure pollen, as it is stuck together with nectar and bee saliva. On the other hand it could be less dense, because air spaces between the grains are included in the volume. The calculations of pollen volume per flower are based on the volume and numbers of individual pollen grains.

There are many sources of uncertainty in these estimates of pollen demand per larva. Some are encompassed to some extent in the range in Tables S3 and S7, others may not be. For bumblebees, the uncertainty captured by the range in Table S3 comes partly from a lack of precise data about the relationship between volume of pollen grains and the mass of corbicular loads, and from the range of values we could find for pollen consumption by

individual larvae (two studies only, both on the *B. terrestris*, one working with whole colonies, one only with males; Table 3.1). Uncertainty about the difference in pollen requirement between bumblebee species is not captured because data were only available for one species. The studies citied in Table S3.1 demonstrate that the volume of pollen required for larval rearing depends on the type of pollen (because different types differ in nutritional quality). This is likely to be true for solitary bees and bumblebees, and is captured to some extent in our bumblebee parameter ranges, but not those for solitary bees. For solitary bees, the range in parameter values in Table S7 comes from uncertainty about body size - we found no data on the dry body mass of individuals of the selected species. Another uncertainty, not captured quantitatively, is that the regression equation underlying the calculations is based on a set of oligolectic, relatively rare solitary bees whose requirements may be different from the common solitary bees for which we develop flower demands.

The reported calculations are based only on pollen demand to rear bee larvae from egg to adult, since these are the available data. They do not take account of the pollen requirement of adults once they are flying, including overwintering queens, which is more difficult to quantify.

Number of bees per nest per month

The total number of workers and reproductives (males and queens) produced by a bumblebee colony varies according to species and environmental conditions. In general, bumblebee colonies are often reported to produce 200 workers or more (Prys-Jones and Corbet 2011), while the number of reproductives (drones and gynes) is highly variable. There are some recent data on the number of individual bees in 60 *Bombus terrestris* colonies placed at three English field sites for 14 to 16 weeks. These colonies were frozen and measured at a point when they were producing reproductives and numbers were starting to decline. The average

number of workers, drones, queens (including pupae) larvae, pupae (drones and workers) and eggs was 396.87 (see Table S4). Although workers from early in the colony cycle have been lost and therefore are not counted, 26% of the individuals that are counted were eggs or larvae at the point of colony destruction, when the colonies had passed their peak. These may never have made it to adulthood. Since *Bombus terrestris* and *B. lapidarius* are both considered species with 'large' colonies (Prys-Jones and Corbet 2011), it seems reasonable to use this value, rounded to 400 for simplicity, as the expected number of bumblebees raised in individual colonies of these species. The same authors list *Bombus pascuorum* as having 'medium' sized colonies. We could not find data to support this, but we have used a lower estimate of 300 individuals per colony to reflect the reported difference in colony size. We include workers as well as reproductives, because we are looking for a floral resource to support rearing for the full colony cycle, and the workers must be raised and fed first, in order to produce queens and males. Of course, queens, males and workers probably have different nutritional requirements, a detail that is ignored here.

To calculate a number of bees being reared by bumblebee colonies per month, we have used phenology data gathered by the Bumblebee Conservation Trust from its nationwide bee monitoring scheme BeeWalk. Table S8 shows the proportions of all individuals (queens, workers and males) counted in each month, for the three focal species in 2013 and 2014.

For solitary bees, we do not have per nest productivity data from the wider environment for any of the focal species, but there is ecological information for other species. For example, Williams and Kremen (Williams and Kremen 2007) measured a per capita offspring production of around three offspring/nest in different types of farmland in California for the cavity nesting solitary bee *Osmia lignaria* (mean and standard errors shown in Figure 3 of Williams and Kremen, 2007; exact numbers not reported). Based on this, we assume three offspring are produced per year by each solitary bee nest. One of the target species, *Andrena*

flavipes, has two generations per year (known as ‘bi-voltine’), so we assume there are twice as many nests as the density data suggest – 2,000/100 ha.

Most solitary bee species have relatively limited flight periods, throughout which they are provisioning nests at fairly uniform rate. We therefore divided the flower demand equally across the months of the flight periods of the solitary bee species.

The uncertainty surrounding the number of bees per colony is difficult to quantify, due to a lack of data. As we only had estimates from one study for one of our focal species, we chose not to capture this uncertainty in the parameter ranges in Table S7.

Number of nests per hectare

To our knowledge, there are no estimates of the density of colonies or the numbers of individual bees required to supply a viable pollination service, and no population modelling for bumblebees or solitary bees that can provide threshold densities or reproductive rates required to retain stable or increasing wild bee populations in farmed landscapes.

For bumblebees, there are a number of estimates of real colony densities for the focal species generated using a molecular genetic approach. These are shown in Table S5. The studies cited were generally carried out in agricultural landscapes of moderate to high heterogeneity. For example, Darvill *et al*. (2004) worked in complex and diverse landscapes of southern England and Dreier *et al*. (2014) worked in an agricultural landscape containing targeted agri-environment measures. The values in Table S5 could therefore be seen as baseline densities, already thriving in existing diverse landscapes. The logic of using them as a basis for calculating resource demand underlying a package of new measures is that the measures proposed will be additional to what is already present in the landscape. Adding this many resources to resource-poor landscapes might be expected to bring wild bee populations up to

the level of these diverse landscapes. Adding them to diverse landscapes might be expected to have less effect because the resources are less limiting. The size of the effect (how many bee species and individuals they actually feed) will depend on the resources available in the surrounding landscape and the degree of ecological contrast between the new resource provision and its immediate surroundings (Scheper et al. 2013).

For the three solitary bee species in our list, we did not find any studies of population size or nest density. Estimates of population density are available for other, much less common, ground-nesting bee species. Since these uncommon bees are reported to be very common where they are found, it seems reasonable to use the same numbers to approximate the density of individual nests that a pollinator conservation strategy should aim to support. Franzen and Nilsson (2010, 2013) measured population density of two *Andrena* species (*Andrena hattorfiana* and *A. humilis*) in an area of farmland with patches of semi-natural grassland in Sweden. In the grassland nesting habitat, the highest number of nesting females observed in any single year can be estimated as between 879 and 1,230 nests/km^2 for these two species respectively (see original papers for variance measures). We have used these numbers to set a target density of 1,000 nests/ 100 ha of our target species (an approximation from the average of these two densities).

For solitary bees, as we had no estimates of nest density for the focal species, we chose not to capture this uncertainty in the parameter ranges in Table S7. For bumblebees, several studies provided estimates for each of the focal species, so we used these to generate high and low estimates in Table S7.

Flowers demands (Equation 2)

Pollen volume per flower

As explained in the main text, pollen volume per floral unit was derived either from data provided by Muller *et al.* (2006) or from a new unpublished dataset. The methods for measuring pollen volume per flower in the latter dataset are summarised below.

To assess the volume of pollen offered by 153 of the most common British plants, we extracted pollen from newly dehiscent stamens. Flower buds of each species were collected in the field from two populations when possible, or one. For each population, after waiting for flowers to open in the lab (24-72h), newly dehiscent stamens were collected, counted (range 1-460 stamens per tube depending on the species) and stored in five 1.5 mL eppedendorf tubes filled with 70% EtOH. The samples were vortexed for 30s and sonicated for 10 min to release the pollen from the anthers and the plant debris were manually removed after four successive rinses with 200-400µL of 70% EtOH. The resultant filtrate was centrifuged for 10 min at rcf 14 to produce a concentrated pellet of pollen which was dried in the oven at 60°C for 30-90 min. The pellet of pollen was re-suspended in a known volume of 70% EtOH (60 to 1000µL depending on the species). The number of pollen grains was quantified by two independent visual counts under light microscopy using a counting chamber composed of 144 squares of 0.0125µL (Haemocytometer of the modified Fuchs-Rosenthal). We also measured the major (ma; µm) and minor (mi; µm) axes of 5 pollen grains from each sample by metric graticulated ocular measurement. The volume of one pollen grain was calculated as the volume of an ellipsoid ($V=1/6*pi*mi^2*ma$; µm3) (Buchmann and Orourke 1991, Dasilveira 1991, Roulston et al. 2000). The volume of pollen per stamen was the product of the number of pollen grains per stamens and the average volume of one pollen grain of this species (Biesmeijer et al. 1992). The volume of pollen per flower was simply scaled-up taking into account of the mean number of stamens per flower, corrected by a dicliny factor (1 for hermaphrodite species; 0.5 for dioecious, gynodioecious, monoecious and gynomonoecious species; 0.75 for trimonoecious species).

As pollen volume per flower is a denominator in Equation 2, a lower average pollen volume per flower (1.83 from Baude dataset) produces a higher demand for flowers, while a higher pollen volume per flower (3.2 from Mueller dataset) produces a lower overall flower demand (Table S7).

As shown in Table S10.1 for three individual plant species, the accuracy of estimations of pollen volume per floral unit in the Baude dataset is relatively low (large standard deviations and small sample sizes), introducing substantial uncertainty. Sample sizes were larger and standard deviations relatively smaller in the Muller *et al*. dataset (2006; Appendix A), but these authors did not measure pollen volumes in the common hedgerow or clover species of interest. This uncertainty in pollen volume per flower is not captured in our high and low parameter range, because we have used mean values across all plant species from the two independent data sets. As mentioned in the main text (Discussion), it is perhaps the most important uncertainty in these calculations, and a priority for future research.

Estimating areas of habitat (Equations 3 and S1)

Table S10 and S11 show how calculations of the habitat area that can supply pollen demands for specific plant and bee communities will be possible in the near future.

Flower density values of *Crataegus monogyna*, *Prunus spinosa* and *Trifolium pratense* were measured by the Agriland project (Baude *et al*, in prep.). Flower density is the number of open flowers per unit of vegetative cover of the species of interest. It was estimated through the ratio between the number of flowers (average number of flowers per floral unit multiplied by the number of floral units counted) and the vegetative cover estimated in 5 quadrats (0.5m x 0.5m) placed on selected flowering patches in two distinct populations. For trees, flower density consists of all the open flowers seen in the column situated above the unit of vegetative cover.

Since *Crataegus monogyna* was represented by high trees (height=3.72 ± 0.34 m), flower density was scaled to represent 2m high hedges.

The two selected hedgerow species in Table S10 flower in different months and are used by long-tongued, short-tongued bumblebees and solitary bees as sources of pollen. Willows (*Salix* sp.) are also a pollen source for bees in spring and prevalent in some landscapes, but at this time there were no available pollen data for willow, so these calculations were not possible.

We used an alternative version of Equation 3 in the main text (Equation S1), to estimate the area of a legume-rich sward with 10% red clover cover (*Trifolium pratense*) that could supply the calculated June and July pollen demand of *Bombus pascuorum*.

Equation S1

$$A = \frac{PD}{PCF \times CH \times 0.1}$$

A is the area of clover-rich sward required per 100 ha per month (ha), *PD* is the total pollen demand (mm^3) per 100 ha per month from Equation 1, *PCF* is the volume of pollen per red clover floral unit (mm^3) and *CH* is the number of red clover floral units per ha of uniform red clover sward, which is multiplied by 0.1 to allow for 10% cover. This equation is used to calculate values in Table S11. For the density of red clover floral units (*CH*), a mean of 169.4 was measured for pure patches of flowering *Trifolium pratense* (Table S10.1; calculated by dividing the floral units/m^2 by the mean number of flowers per floral unit). This falls in the range of 50-250 reported by Everaars and Dormann (Everaars and Dormann 2014). The pollen volume per floral unit (PCF) is calculated by multiplying the measured pollen volume per individual flower by the mean number of flowers per floral unit (values given in Table S10.1).

The substantial uncertainty in estimated floral unit densities, especially for the two hedgerow species (see standard deviations for flowers/m^2, Table S10.1), is not captured in our estimates

of habitat areas required to meet estimated pollen demands. For red clover, uncertainty is somewhat lower because the empirical estimate is supported by other studies.

Table S1 A proposed set of declining bee species found in moderately intensive farmland, and their ecological requirements. This list was developed in August 2014 as an initial proposal for spatial targeting of the Wild Pollinator and Farm Wildlife Package, by a Working Group established by Natural England. The information below is based on expert knowledge and unpublished analyses of records held by the Bees Wasps and Ants Recording Society. The Working Group selected bee species found on farmland, with well-known ecology and distribution, which are either **declining** according to a new (incomplete) Red List analysis or **have declined** prior to the Red List focal period and are known to benefit from agri-environment options (the latter applies only to *Bombus ruderatus*). As for the dominant pollinators in Table 2, all the identified species are ground-nesting. Cavity-nesting species tend to be associated more with built up areas, scrub or woodland.

Species	Forage requirements	Nesting requirements
Bombus ruderarius (Red-shanked carder bee)	Legumes, especially *Lotus corniculatus*	On surface of open grassland.
Bombus ruderatus (Large garden bumblebee)	Legumes, especially *Trifolium pratense*, but also *Symphytum officinale* and *Iris pseudacorus* early in year.	In tall grasses with small mammal nests.
Andrena varians	Often associated with rosaceous shrubs and trees.	In ground, probably bare or short turf.
Andrena fucata	A variety of spring-flowering species, herbaceous and woody.	In ground, probably bare or short turf.
Andrena fulva	Often associated with rosaceous shrubs and trees.	In ground, probably bare or short turf.
Andrena denticulata	Only collects pollen at yellow Asteraceae.	In ground, probably bare or short turf.
Andrena fulvago	Only collects pollen at yellow Asteraceae.	In ground, probably bare or short turf.
Andrena humilis	Only collects pollen at yellow Asteraceae.	In ground, probably bare or short turf.
Eucera longicornis	Only collects pollen at Fabaceae, notably *Lathyrus* species.	Inclined bare ground, with a clay component.
Andrena hattorfiana	Only collects pollen at *Knautia arvensis* or in some cases *Centaurea scabiosa* in the absence of *Knautia*.	In ground, using bare ground or short turf.
Andrena marginata	Only collects pollen at *Scabiosa columnaria* or *Succisa pratensis* (apparently not on calcareous soils, even though the plant will grow there).	In ground, using bare ground or short turf.
Melitta leporina	Only collects pollen at Fabaceae.	In ground, using bare ground or short turf.

Table S2 Interventions that could benefit UK wild bee populations, scored for importance by conservation practitioners, for certainty by experts (based on evidence summarised in Dicks *et al.* (2010)) and ranked according to an Advocacy Priority Index based on the geometric distance from the point of maximum importance, maximum certainty - the lower the distance, the higher the advocacy priority (Equation S2). Methods are described by Sutherland *et al.* (Sutherland et al. 2011, Sutherland et al. 2012), although some of these data are previously unpublished. Ranks are given for the top ten interventions applicable in farmed landscapes, and these are included in Table 4. Advocacy Priority Index and rank are only shown if evidence supports the intervention being effective.

Rank (farmland only)	Intervention	Certainty Score	Importance Score	Advocacy Priority Index[1]
1	Sow uncropped arable field margins with a native wild flower seed mix	53.33	41.43	1.74
2	Restore species-rich grassland vegetation	36.67	51.36	1.52
	Plant parks and gardens with appropriate flowers	46.67	37.57	1.50
3	Plant dedicated floral resources on farmland[2]	61.67	24.09	1.38
4	Introduce agri-environment schemes generally[2]	30.00	45.91	1.34
5	Sow uncropped arable field margins with an agricultural nectar and pollen mix	55.00	22.16	1.27
6	Create patches of bare ground for ground-nesting bees	55.00	20.52	1.24
7	Restore lowland heathland	45.00	20.45	1.15
	Leave field margins unsprayed within the crop (conservation headlands)[3]	50.00	17.43	
8	Provide set-aside areas in farmland	56.67	10.84	1.07
	Restore species-rich grassland on road verges	13.33	40.80	1.07
9	Provide artificial nest sites for solitary bees	81.67	4.84	1.06
10	Leave arable field margins uncropped with natural regeneration	53.33	10.30	1.05
	Provide artificial nest sites for bumblebees[3]	71.67	3.93	
	Increase the diversity of nectar and pollen plants in the landscape	6.00	41.89	1.00
	Increase the proportion of natural habitat in the farmed landscape	0.00	62.32	1.00
	Convert to organic farming	45.00	8.93	0.98
	Protect existing natural or semi-natural habitat to prevent conversion to agriculture	0.00	46.82	0.97
	Reduce intensity of farmland meadow management	38.33	10.84	0.97
	Connect areas of natural habitat together	0.00	46.27	0.97
	Manage hedges to benefit bees	28.33	15.50	0.96
	Provide training to conservationists and land managers on bee ecology and conservation	0.00	44.43	0.96
	Practise/encourage 'wildlife gardening'	8.33	31.55	0.96
	Increase the use of clover leys on farmland	9.33	28.68	0.95
	Provide grass strips at field margins	48.33	3.43	0.93
	Rear declining bumblebees in captivity	51.67	1.68	0.92

**Rank (farmland only)**	**Intervention**	**Certainty Score**	**Importance Score**	**Advocacy Priority Index**[1]
	Restrict certain pesticides	10.00	24.05	0.92
	Raise awareness amongst the general public through campaigns and public information	0.00	32.50	0.90
	Reduce pesticide or herbicide use generally	4.00	27.27	0.90
	Protect brownfield sites	0.00	30.34	0.89
	Reduce grazing intensity on pastures	18.00	13.34	0.88
	Increase areas of rough grassland for bumblebee nesting	23.33	8.91	0.87
	Prevent escape of commercial bumblebees from greenhouses	26.67	6.32	0.86
	Manage land under power lines for wildlife	6.67	16.70	0.84
	Reintroduce laboratory-reared bumblebee colonies to the wild	31.67	1.75	0.84
	Eradicate threatening non-native bees or bee parasites	8.33	14.25	0.83
	Keep pure breeding populations of native honey bee subspecies	18.33	6.02	0.82
	Rear and manage solitary bee populations	26.67	1.64	0.82
	Ensure commercial hives/nests are disease free	4.00	14.34	0.81
	Enhance bee taxonomy skills through higher education and training	0.00	17.05	0.81
	Retain dead wood in forest management	0.00	16.41	0.81
	Re-plant native forest	0.00	15.98	0.80
	Reduce tillage	15.00	5.34	0.80
	Reduce fertilizer run-off into margins	0.00	13.05	0.78
	Legally protect large native trees	5.00	6.48	0.77
	Prevent spread of the small hive beetle	4.33	6.48	0.76
	Translocate bumblebee colonies in nest boxes	11.67	1.36	0.76
	Control deployment of non-native hives/nests	0.00	8.00	0.75
	Control fire risk using mechanical shrub control and/or prescribed burning	5.00	4.41	0.75
	Introducing mated females to small populations to improve genetic diversity	3.33	3.57	0.74
	Conserve old buildings or structures as nesting sites for bees	0.00	5.45	0.74
	Translocate solitary bees	6.00	1.48	0.74
	Reintroduce laboratory-reared bumblebee queens to the wild	0.00	1.80	0.72
	Exclude bumblebee nest predators (such as badgers) from sensitive sites	0.00	1.75	0.72

[1] Advocacy Priority Index was calculated using the following equation:

Equation S2

$$API = \frac{1}{\sqrt{\left(\left(100 - i\left(\frac{100}{i_{\max}}\right)\right)^2 + (100 - c)^2\right)}}$$

Where *API* = Advocacy Priority Index, *c* = certainty score, *i* = importance score, i_{max}=maximum certainty score across all interventions (used to generate a standardised importance score).

[2] Excluded from Table 3 (main text), as too general to be relevant for agri-environment package design.

[3] For these interventions, Advocacy Priority Index is not shown, as the relatively high certainty scores reflect evidence showing the intervention is NOT effective for bumblebees, something those scoring for importance may not have known. This demonstrates a flaw in the method that has since been addressed in subsequent exercises (Dicks *et al*. in prep) by combining scores for effectiveness and certainty into a single categorical scale.

Table S3 Estimates of pollen required per larva. Information on flowers visited is taken from the BWARS website (http://www.bwars.com). Body length information is gleaned from a range of websites following a Google search. Ranges of pollen requirement per larvae for specialist solitary bees of these sizes were calculated by Muller *et al* (2006). Pollen requirements for bumblebee larvae are from data shown in Table S3.1.

**Species**	**Adult body length estimate (mm)**	**Pollen required/larva for bees of this size (mm^3)**	**Flowers visited**
Andrena flavipes	10-14	30.24 – 75.49	Many open flowers, including hedgerow species such as bramble and hawthorn, and fruit trees.
Andrena haemorrhoa	8-11	17.19 – 34.59	Open flowers, including hedgerow species such as bramble and hawthorn, and fruit trees.
Andrena cineraria	10-15	30.24 – 75.49	Brambles (*Rubus* spp.), cabbages (*Brassica* spp.), cherries (including blackthorn (*Prunus spinosa*) and wild cherry (*Prunus avium*)), dandelion (*Taraxacum officinale*), daisies (*Bellis perennis*), gorse (*Ulex europaea*), hogweed (*Heracleum sphondylium*), pears (*Pyrus* spp.), plum (*Prunus* sp.), thrift (*Armeria maritima*), willows (*Salix* spp.) and wood spurge (*Euphorbia amygdaloiodes*).
Bombus sp. (long tongued)		210-772	Associated with flowers with longer corollae, especially Fabaceae and Scrophulariacae.
Bombus sp. (short-tongued)		210-772	Visits are made to a wide variety of flowers, both for pollen and nectar.

Table S3.1 Estimates of pollen demand per larvae in mm^3 for *Bombus terrestris*. Means (standard errors) are given for reported data. These measures represent mass and volume of commercially available honey-bee collected 'pollen' (derived from corbicular loads), rather than of actual pollen grains. Masses (mg) and volumes (mm^3) rounded to 1 decimal place. Density estimates are from Table S3.2. Values in bold are the high and low end of the range, used to estimate the range of flower demands.

**Study/pollen diet**	**Number of colonies (n)**	**Pollen (g) consumed per colony**	**Larvae produced per colony**	**Pollen mass /larva (mg)**	**Pollen volume / larva (mm^3)** Assuming density =1.09 mg/mm^3	**Pollen volume / larva (mm^3)** Assuming density =1.7 mg/mm^3
Genissel *et al.* (2002)[1] *Salix*-dominated pollen diet	5	6.9 (1.1)	8.2 (2.8)	841.5	**772.0**	495.0
Genissel *et al.* (2002)[1] *Prunus*-dominated pollen diet	5	8.2 (0.2)	19.2 (1.7)	427.1	391.8	251.2
Genissel *et al.* (2002)[1] Blended pollen diet	5	8.9 (1.4)	13.6 (3.3)	654.4	600.4	384.9
Ribeiro et al (1996)[2] Fresh frozen pollen	8	80	170	470.5	431.7	276.8
Ribeiro et al (1996)[2] Dried frozen pollen	7	50	140	357.1	327.7	**210.1**

[1] Experiments using worker micro-colonies; all larvae male.

[2] Numbers include queens, males and workers, but approximated from Figures. Raw data unavailable.

Table S3.2 Estimates of the mass and density of honey bee *Apis mellifera* corbicular loads. Data on corbicular mass derived from Roman (2006), Tables 2 and 3. The estimates of corbicular volume are derived from corbicular area measurements in Milne *et al*. (1986) – see text. Values in bold are the high and low end of the range, used in Table S3.1.

Year	Mean mass per corbicular load (mg)	Standard deviation	Number of colonies	Number of sampling events (once every three days)	Estimated density (mg/mm^3) assuming volume = 4 mm^3	Estimated density (mg/mm^3) assuming volume = 6 mm^3
2004	6.54	1.13	19	28	1.635	**1.09**
2005	6.80	1.19	19	28	**1.7**	1.133

Table S4 Number of bumblebee individuals counted in 60 free-living, boxed *Bombus terrestris* colonies, reared in a laboratory and placed at three agricultural sites in Lincolnshire or Yorkshire, in April 2012, as the first workers were emerging. Colonies were frozen and bees counted after 14-16 weeks, when reproductives were being produced and colonies had started to decline (as shown by decreasing mass). Mean and (standard error) for the numbers of individuals per colony in each category are given, with samples pooled across three experimental sites (n=60) that differed in the chemical treatment of oilseed rape fields within 1 km. Source: Food and Environment Research Agency (2013), raw data supplied by Helen Thompson.

Queens (including pupae)	Workers	Drones	Eggs	Larvae	Pupae	Total
25.98 (4.79)	39.43 (5.07)	48.27 (6.48)	33.63 (7.33)	70.92 (8.46)	179.20 (16.58)	396.87 (32.39)

Table S5 Published estimates of nest density in agricultural landscapes for the six focal bee species

For studies that made estimates for more than one landscape, mean estimates (standard errors) are given. Values in bold are the high and low end of the range for each species, used to estimate the range of flower demands.

Species	Nest density estimates/100 ha	Number of landscapes (*n*)	Source
Bombus terrestris	**13**	1	Darvill *et al.* 2004
	13.89	1	Dreier *et al*. 2014
	25.97 (1.18)	6	Huth-Schwarz *et al.* 2012
	28.7	1	Knight *et al*. 2005
	**78.67** (13.81)	12	Stanley *et al*. 2013
Bombus pascuorum	**7.85** (0.61)	13	Herrmann *et al.* 2007
	18.95	1	Dreier *et al*. 2014
	34.91 (3.87)	10	Knight *et al.* 2009; foraging range 1 km
	67.8	1	Knight *et al*. 2005
	173.21 (19.19)	10	Knight *et al.* 2009; foraging range 450 m
	**193**	1	Darvill *et al.* 2004; low foraging range assumed
Bombus lapidarius	**35.16**	1	Dreier *et al.* 2014
	81.14 (10.94)	7	Stanley *et al*. 2013
	**117.2**	1	Knight *et al*. 2005
Andrena flavipes	NONE FOUND		
Andrena haemorrhoa	NONE FOUND		
Andrena cineraria	NONE FOUND		

Table S6 Summary data for pollen quantity (mm^3) per floral unit, measured by Muller *et al.* (2006) and Baude *et al.* (in prep.). For the Baude data, mean volume of pollen per flower is corrected by a "dicliny factor" (1 for hermaphrodite species; 0.5 for dioecious, gynodioecious, monoecious and gynomonoecious species; 0.75 for trimonoecious species). Plant species lists are provided below. * = species for which the floral unit was an inflorescence rather than a single flower. Species names in bold are included in both datasets.

	Muller *et al* (2006)[1]	Baude *et al* (unpublished)[2]
Number of plant species	16	153
Mean volume pollen per floral unit (standard error) mm^3	3.20 (1.51)	1.83 (0.35)

[1] Species included: *Buphthalmum salicifolium**, *Campanula patula*, ***Campanula rotundifolia***, ***Convolvulus arvensis***, *Echium vulgare*, *Erysimum rhaeticum*, ***Hedera helix***, ***Knautia arvensis****, ***Lotus corniculatus***, ***Lythrum salicaria***, ***Medicago sativa***, *Onobrychis viciifolia*, ***Ranunculus acris***, *Reseda lutea*, *Stachys recta*, ***Succisa pratensis****

[2] Species included: *Acer pseudoplatanus, Achillea millefolium*, Aesculus hippocastanum, Agrimonia eupatoria, Ajuga reptans, Allium cepa*, Anagallis arvensis, Anemone nemorosa, Angelica sylvestris*, Anthriscus sylvestris*, Arctium* agg.**, Armeria maritima*, Aster tripolium*, Bellis perennis*, Brassica napus, Brassica rapa*, Buddleja davidii*, Calluna vulgaris, Caltha palustris, Calystegia sepium,* ***Campanula rotundifolia****, Capsella bursa-pastoris, Cardamine pratensis, Castanea sativa*, Centaurea nigra*, Cerastium fontanum, Chamerion angustifolium, Circaea lutetiana ,Cirsium acaule*, Cirsium arvense*, Cirsium palustre*, Cirsium vulgare*, Clematis vitalba,* ***Convolvulus arvensis****, Cornus sanguinea, Corylus avellana*, Crataegus monogyna, Crepis capillaris*, Cytisus scoparius, Daucus carota*, Digitalis purpurea, Epilobium hirsutum, Epilobium montanum, Epilobium tetragonum, Erica cinerea, Erica tetralix, Eupatorium cannabinum*, Euphrasia officinalis* agg.*, Fallopia convolvulus, Fallopia japonica, Filipendula ulmaria*, Galium aparine, Galium mollugo, Galium verum*, Geranium molle, Geranium pratense, Geum urbanum, Gladiolus* sp.*, Glechoma hederacea,* ***Hedera helix*****, Helianthemum nummularium, Heracleum sphondylium*, Hyacinthoides non-scripta, Hypericum pulchrum, Hypochaeris radicata*, Impatiens glandulifera, Iris pseudacorus,* ***Knautia arvensis*****, Lamiastrum galeobdolon, Lamium album, Lamium purpureum, Lapsana communis*, Lathyrus latifolius, Lathyrus pratensis, Leontodon autumnalis*, Leontodon hispidus*, Leucanthemum vulgare*, Ligustrum vulgare*, Linaria vulgaris, Lonicera periclymenum, Lotus corniculatus, Lotus pedunculatus, Lysimachia nemorum,* ***Lythrum salicaria****, Malus domestica, Matricaria discoidea*, Matricaria recutita*, Medicago lupulina*,* ***Medicago sativa****, Mercurialis perennis, Myosotis arvensis, Odontites vernus, Origanum vulgare, Oxalis acetosellam, Papaver rhoeas, Pastinaca sativa*, Picris echioides*, Pilosella officinarum*, Pisum sativum, Plantago coronopus*, Plantago lanceolata*, Plantago major*, Polygonum aviculare agg., Potentilla anserine, Primula vulgaris, Prunella vulgaris, Prunus avium, Prunus spinosa, Pulicaria dysenterica*,* ***Ranunculus acris****, Ranunculus bulbosus, Ranunculus ficaria, Ranunculus flammula, Ranunculus repens, Raphanus raphanistrum, Raphanus sativus, Rhinanthus minor, Rosa canina agg., Rubus fruticosus agg., Sagina sp., Sambucus nigra, Saponaria officinalis, Senecio jacobaea*, Senecio vulgaris*, Silene dioica, Silene vulgaris, Sinapis arvensis, Sisymbrium officinale*, Sium latifolium*, Solanum dulcamara, Solanum nigrum, Solanum tuberosum, Sonchus arvensis*, Sonchus asper*, Sonchus oleraceus*, Sorbus aucuparia, Stachys sylvatica, Stellaria graminea,* ***Succisa pratensis*****, Taraxacum* agg.**, Teucrium scorodonia, Tilia platyphyllos, Trifolium dubium*, Trifolium pratense*, Trifolium repens*, Ulex europaeus, Vaccinium myrtillus, Veronica chamaedrys, Veronica persica, Veronica serpyllifolia, Vicia cracca, Vicia sativa, Viola riviniana/reichenbiana*

Table S7 Figures underlying high and low flower demand estimates per 100 ha for six dominant crop pollinating bee species shown in Figures 1 and 2. This Table is to show how maximum and minimum estimates for each parameter were used to illustrate the range of possible flower demands across the whole season or activity period of the colony. Numbers of floral units in the final column are rounded to integers.

Species	**Pollen/larva (mm^3)[1]**	**Bees reared /colony or nest[2]**	**Nests or colonies /100ha[3]**	**Bees reared /100ha**	**Average volume pollen /floral unit (mm^3)[4]**	**Floral units needed for larval rearing/100ha[5]**
**LOW estimates**						
Andrena flavipes	30.24	3	2000	6000	3.2	56,700
Andrena haemorrhoa	17.19	3	1000	3000	3.2	16,115
Andrena cineraria	30.24	3	1000	3000	3.2	28,350
Bombus lapidarius	210	400	35	14000	3.2	918,750
Bombus pascuorum	210	300	8	2400	3.2	157,500
Bombus terrestris	210	400	13	5200	3.2	341,250
**HIGH estimates**						
Andrena flavipes	75.49	3	2000	6000	1.83	247,508
Andrena haemorrhoa	34.59	3	1000	3000	1.83	56,705
Andrena cineraria	75.49	3	1000	3000	1.83	123,754
Bombus lapidarius	772	400	117	46800	1.83	19,742,950
Bombus pascuorum	772	300	193	57900	1.83	24,425,574
Bombus terrestris	772	400	79	31600	1.83	13,330,710

[1] See Tables S3 and S3.1
[2] See Table S4 (bumblebees) and methods text (bumblebees and solitary bees)
[3] See Table S5 and main text
[4] See Table S6 for sources.
[5] Calculated according to Equations 1 and 2, main text.

Table S8. Proportion of total individuals expected in each month of the year for the six dominant crop pollinating bee species. For solitary bees information on flight period comes from the bee traits database maintained by S.P.M. Roberts at the University of Reading, UK. It was established by the ALARM project (www.ALARM-project.net) and further developed by the STEP project (www.STEP-project.net). Numbers of bees are assumed to be equally distributed across the active flying months. For bumblebees, this is based on the proportion of all individuals recorded in each month of the year, from BeeWalk data collected in 2013 and 2014 on 245 transects in the UK. These numbers are rounded to 2 decimal places. Source (bumblebees): Richard Comont, Bumblebee Conservation Trust.

**Species**	**Jan**	**Feb**	**Mar**	**Apr**	**May**	**Jun**	**Jul**	**Aug**	**Sep**	**Oct**	**Nov**	**Dec**
Andrena flavipes	0	0	0.125	0.125	0.125	0.125	0.125	0.125	0.125	0.125	0	0
Andrena haemorrhoa	0	0	0.2	0.2	0.2	0.2	0.2	0	0	0	0	0
Andrena cineraria	0	0	0.2	0.2	0.2	0.2	0.2	0	0	0	0	0
Bombus lapidarius	0.00	0.00	0.01	0.04	0.04	0.25	0.32	0.32	0.02	0.00	0.00	0.00
Bombus pascuorum	0.00	0.00	0.01	0.03	0.08	0.17	0.19	0.37	0.13	0.02	0.00	0.00
Bombus terrestris	0.00	0.00	0.05	0.10	0.08	0.22	0.26	0.24	0.04	0.01	0.00	0.00

*NOTE: a few species, including *Bombus terrestris* and *Bombus pascuroum*, are sometimes seen flying and even forming colonies in the winter months. For example, Stelzer *et al.* (2010) documented winter active *B. terrestris* colonies in urban areas of the UK. While this phenomenon may be increasing, it is not included here. The BeeWalk data has a very small proportion (0.03%, or 4 inidividuals) of *B. pascuorum* recorded in January and November. These have been removed from the proportions used in this paper.

Table S9 Calculated combined pollen demand for larval rearing, in floral units/100 ha/month, for six dominant crop-pollinating wild bee species. Months not shown (Jan, Feb, Nov and Dec) have no pollen demand, because they are outside the normal flight period of these and all other bee species (but see note under Table S8). Upper and lower bounds are given, based on values given in Tables S7 and S8, to show the range of uncertainty. Numbers for solitary bees are the sums of floral unit demands in each month for three solitary bee species *Andrena flavipes*, *Andrena haemorrhoa* and *Andrena cineraria*. Short-tongued bumblebees are the sums of floral unit demands in each month for the two species *Bombus terrestris* and *B. lapidarius*. Long-tongued bumblebees represented by *B. pascuorum*. Numbers are rounded to integers. Low estimates are shown in Figure 1.

	**Mar**	**Apr**	**May**	**Jun**	**Jul**	**Aug**	**Sep**	**Oct**
**LOW estimates**								
Solitary bees	15981	15981	15981	15981	15981	7088	7088	7088
Short-tongued bumblebees	23329	68801	62942	309645	383017	377407	30016	4844
Long-tongued bumblebees	1327	4046	12913	26486	30565	58164	20632	3367
TOTAL	40636	88828	91836	352111	429563	442658	57736	15298
**HIGH estimates**								
	**Mar**	**Apr**	**May**	**Jun**	**Jul**	**Aug**	**Sep**	**Oct**
Solitary bees	67030	67030	67030	67030	67030	30939	30939	30939
Short-tongued bumblebees	782300	2102264	1832785	7979188	9765405	9543857	888207	179656
Long-tongued bumblebees	205735	627543	2002554	4107458	4740170	9020280	3199744	522091
TOTAL	1055065	2796837	3902369	12153677	14572605	18595076	4118889	732685

Table S10 Estimating the length of hedgerow - an illustration of more detailed calculations for specific flowering species. The species-specific flower density and pollen volume data were measured by Baude *et al*. (see Supplementary Materials and Methods; Table S10.1). Floral units are individual flowers for both species. Pollen demand for the appropriate month is calculated using Equation 1 (main text), numbers from Table S7 and S8, summed across all six dominant pollinator species and rounded to the nearest mm^3. The number of metres of hedge is calculated using Equation 3 (main text), and presented to the nearest metre.

**Hedgerow species**	**Flowering period**	***Floral units/m²**	**Pollen volume/ floral unit (mm³)**	**Total pollen demand (mm³ /100ha) LOW**	**Total Pollen demand (mm³ /100ha) HIGH**	**Metres of single species hedgerow / 100 ha LOW**	**Metres of single species hedgerow/ 100 ha HIGH**
Prunus spinosa	April	1327.88	0.28	284,250	5,118,211	765	13,766
Crataegus monogyna	May	5584.78	0.33	293,874	7,141,335	159	3,863

* Flower density was adjusted to represent hedges 2 m high x 1 m wide.

Table S10.1 Flower densities and pollen volumes for three species in Tables S10 and S11

	**Flowers/ floral unit**		**Flowers/m²**		**Pollen volume/flower(mm³)[1]**	
	**Mean**	**Standard deviation**	**Mean**	**Standard deviation**	**Mean**	**Standard deviation**
Prunus spinosa	1	0	1327.88	643.75	0.280	0.0956
Crataegus monogyna	1	0	10387.69	11681.96	0.331	0.256
Trifolium pratense	24.1	11.50	4082.21	1081.21	0.00288	0.00280

[1] Standard deviations for pollen volume/floral unit are very high because there are many steps in the calculation, leading to error propagation (pollen number per stamen, volume of one pollen grain, number of stamens per flower, number of flowers per floral unit). Values shown to 3 significant figures.

Table S11 Estimating the area of grass swards with 10% red clover *Trifolium pratense* required to supply June and July pollen demands of *Bombus pascuorum*. The species-specific flower density and pollen volume data were measured by Baude *et al*. (see Supplementary Materials and Methods; Table S10.1). The floral unit for *T. pratense* is a single globular raceme. Pollen demand for the appropriate month is calculated using Equation 1 (main text), numbers from Table S7 and S8, for *Bombus pascuorum* only. The number of hectares of clover-rich sward is calculated using Equation S1, with floral units/m^2 multiplied by 10,000 to calculate the parameter *CH*.

Plant species	Flowering period	Floral units/m^2	Pollen volume/ floral unit (mm^3)	Total pollen demand (mm^3 /100ha) LOW	Total Pollen demand (mm^3 /100ha) HIGH	Ha of 10% clover sward / 100 ha LOW	Ha of 10% clover sward / 100 ha HIGH
Trifolium pratense	June	169.4	0.0694	84,754	7,393,425	7.21	628.89
Trifolium pratense	July	169.4	0.0694	97,809	8,532,306	8.32	725.76

Table S12 Foraging ranges for dominant crop pollinator species.

Species	Estimated from intertegular distance[1]	Measured by Carvell *et al*. (2014)	
	Maximum foraging range (km)	Maximum foraging range (m)	Mean foraging distance (m) (SE)
Andrena flavipes	0.94		
Andrena haemorrhoa	1.06		
Andrena cineraria	1.35		
Bombus lapidarius	2.74	1959.50	549.16 (16.74)
Bombus pascuorum	2.49	1807.50	343.18 (18.32)
Bombus terrestris	3.49	2051.33	552.78 (30.91)

[1] From bee traits database maintained by Stuart Roberts at the University of Reading, UK, established by the ALARM project (www.ALARM-project.net) and further developed by the STEP project (www.STEP-project.net). Foraging ranges calculated from intertegular distance (distance between wing bases) following an equation derived from empirical observations by Greenleaf *et al*. (2007).

Table S13 An overview of uncertainty in the parameters for Equations 1, 2, 3 and S1. There are no entries in the final column for *FM* and *CH* as these parameters were not used in Equations 1 and 2

**Parameter**	**Description**	**Qualitative assessment of uncertainty (high, medium or low)**	**Sources of uncertainty**	**Captured in high and low ranges in Tables S7 and S9 and Figure 1 (yes, no or partially)**
PB	Pollen consumed by individual bee larvae (mm^3)	Medium	Unclear relationship between pollen mass (measured for bees) and pollen volume (measured for flowers)	Yes
			Variation between bee species	Bumblebees: No<br>Solitary bees: Partially
			Variation between castes and sexes	Partially
			Variable nutritional value of different pollen	Bumblebees: Partially<br>Solitary bees: No
BN	Number of new individual bees per month raised in a single colony or nest	Bumblebees: Medium<br><br>Solitary bees: High	Variation between species (parameter estimated based on one bumblebee species, one proxy solitary bee species with a different nesting habit).	No
			Dependence on environmental conditions (including landscape and weather)	No
N	Number of nests per 100 ha	Bumblebees: Low	Variation between species	Bumblebees: Yes<br>Solitary bees: No
		Solitary bees: High	Variation between landscapes	Bumblebees: Yes<br>Solitary bees: No
PF<br>*PHF*<br>*PCF*	Average pollen volume (mm^3) per floral unit	Medium<br><br>Hedgerow species (PHF): High	Variation between species	No
			Variation within species	Partially
			Dependence on environmental conditions	No
FM	Number of floral units per metre of hedge	High	Variation within species	
			Variation between places	

Parameter	Description	Qualitative assessment of uncertainty (high, medium or low)	Sources of uncertainty	Captured in high and low ranges in Tables S7 and S9 and Figure 1 (yes, no or partially)
CH	Number red clover floral units per ha of uniform clover sward	Low	Variation within species	
			Variation between places	

References

Biesmeijer, J. C., B. Vanmarwijk, K. Vandeursen, W. Punt, and M. J. Sommeijer (1992) Pollen Sources for Apis-Mellifera L (Hym, Apidae) in Surinam, Based on Pollen Grain Volume Estimates. *Apidologie*, **23**, 245-256.

Buchmann, S. L., and M. K. Orourke (1991) Importance of Pollen Grain Volumes for Calculating Bee Diets. *Grana*, **30**, 591-595.

Carvell, C., S. Dreier, J. W. Redhead, A. F. G. Bourke, W. C. Jordan, S. Sumner, J. Wang, and M. S. Heard (2014) *Investigating the impact of habitat structure on queen and worker bumblebees in the field.* End of Insect Pollinators Initiative Project Report. BBSRC Grant reference BB/I000925/1, 31pp.

Darvill, B., M. E. Knight, and D. Goulson (2004) Use of genetic markers to quantify bumblebee foraging range and nest density. *Oikos*, **107**, 471-478.

Dasilveira, F. A. (1991) Influence of Pollen Grain Volume on the Estimation of the Relative Importance of Its Source to Bees. *Apidologie*, **22**, 495-502.

Dicks, L. V., D. A. Showler, and W. J. Sutherland (2010) *Bee Conservation: Evidence for the effects of interventions*. 1 edition. Pelagic Publishing.

Dreier, S., J. W. Redhead, I. A. Warren, A. F. G. Bourke, M. S. Heard, W. C. Jordan, S. Sumner, J. L. Wang, and C. Carvell (2014) Fine-scale spatial genetic structure of common and declining bumble bees across an agricultural landscape. *Molecular Ecology*, **23**, 3384-3395.

Everaars, J., and C. F. Dormann (2014) Simulation of solitary (non-*Apis*) bees competing for pollen. In: *In Silico Bees*. CRC Press.

Food and Environment Research Agency (2013) *Effects of neonicotinoid seed treatments on bumble bee colonies under field conditions.* Food and Environment Research Agency, Sand Hutton, York. Available from: http://fera.co.uk/ccss/documents/defraBumbleBeeReportPS2371V4a.pdf.

Franzen, M., and S. G. Nilsson (2010) Both population size and patch quality affect local extinctions and colonizations. *Proceedings of the Royal Society B-Biological Sciences*, **277**, 79-85.

Franzen, M., and S. G. Nilsson. 2013. High population variability and source-sink dynamics in a solitary bee species. *Ecology*, **94**, 1400-1408.

Genissel, A., P. Aupinel, C. Bressac, J. N. Tasei, and C. Chevrier (2002) Influence of pollen origin on performance of *Bombus terrestris* micro-colonies. *Entomologia Experimentalis Et Applicata*, **104**, 329-336.

Greenleaf, S. S., N. M. Williams, R. Winfree, and C. Kremen (2007) Bee foraging ranges and their relationship to body size. *Oecologia*, **153**, 589-596.

Herrmann, F., C. Westphal, R. F. A. Moritz, and I. Steffan-Dewenter (2007) Genetic diversity and mass resources promote colony size and forager densities of a social bee (*Bombus pascuorum*) in agricultural landscapes. *Molecular Ecology*, **16**, 1167-1178.

Huth-Schwarz, A., J. Settele, R. F. A. Moritz, and F. B. Kraus (2012) Factors influencing *Nosema bombi* infections in natural populations of *Bombus terrestris* (Hymenoptera: Apidae). *Journal of Invertebrate Pathology*, **110**, 48-53.

Knight, M. E., A. P. Martin, S. Bishop, J. L. Osborne, R. J. Hale, R. A. Sanderson, and D. Goulson (2005) An interspecific comparison of foraging range and nest density of four bumblebee (*Bombus*) species. *Molecular Ecology*, **14**, 1811-1820.

Knight, M. E., J. L. Osborne, R. A. Sanderson, R. J. Hale, A. P. Martin, and D. Goulson (2009) Bumblebee nest density and the scale of available forage in arable landscapes. Insect *Conservation and Diversity*, **2**, 116-124.

Milne, C. P., R. L. Hellmich, and K. J. Pries (1986) Corbicular Size in Workers from Honeybee Lines Selected for High or Low Pollen Hoarding. *Journal of Apicultural Research* **25**, 50-52.

Muller, A., S. Diener, S. Schnyder, K. Stutz, C. Sedivy, and S. Dorn (2006) Quantitative pollen requirements of solitary bees: Implications for bee conservation and the evolution of bee-flower relationships. *Biological Conservation*, **130**, 604-615.

Prys-Jones, O., and S. Corbet (2011) *Bumblebees*. Pelagic Publishing, Exeter, UK.

Regali, A., and P. Rasmont (1995) New bioassays to evaluate diet in *Bombus terrestris* (L) (hymenoptera, apidae). *Apidologie*, **26**, 273-281.

Ribeiro, M. F., M. J. Duchateau, and H. H. W. Velthuis (1996) Comparison of the effects of two kinds of commercially available pollen on colony development and queen production in the bumble bee *Bombus terrestris* L (Hymenoptera, Apidae). *Apidologie*, **27**, 133-144.

Roman, A. (2006) Effect of pollen load size on the weight of pollen harvested from honeybee colonies. *Journal of Apicultural Science*, **50**, 47-57.

Roulston, T. H., J. H. Cane, and S. L. Buchmann (2000) What governs protein content of pollen: Pollinator preferences, pollen-pistil interactions, or phylogeny? *Ecological Monographs*, **70**, 617-643.

Scheper, J., A. Holzschuh, M. Kuussaari, S. G. Potts, M. Rundlöf, H. G. Smith, and D. Kleijn (2013) Environmental factors driving the effectiveness of European agri-environmental measures in mitigating pollinator loss – a meta-analysis. *Ecology Letters*, **16**, 912-920.

Stanley, D. A., M. E. Knight, and J. C. Stout (2013) Ecological Variation in Response to Mass-Flowering Oilseed Rape and Surrounding Landscape Composition by Members of a Cryptic Bumblebee Complex. *PLoS One*, **8**, e65516.

Stelzer, R. J., L. Chittka, M. Carlton, and T. C. Ings (2010) Winter Active Bumblebees (*Bombus terrestris*) Achieve High Foraging Rates in Urban Britain. *Plos One*, **5**, e9559.

Sutherland, W. J., D. Goulson, S. G. Potts, and L. V. Dicks (2011) Quantifying the impact and relevance of scientific research. *Plos One*, **6(11),** e27537.

Sutherland, W. J., I. Hodge, J. Pretty, and L. V. Dicks (2012) *Testing a Novel Method for Integrating Research, Policy and Practice to Identify Solutions and Research Priorities*. Full Research Report, ESRC End of Award Report, RES-240-25-0006, ESRC, Swindon, UK.

Williams, N. M., and C. Kremen (2007) Resource distributions among habitats determine solitary bee offspring production in a mosaic landscape. *Ecological Applications*, **17**, 910-921.
